# Integrating computed tomography image features improves clinical prediction models for outcomes in nasopharyngeal carcinoma patients treated with (chemo)radiation

**DOI:** 10.1016/j.phro.2026.101040

**Published:** 2026-07-13

**Authors:** Guanzhi Zhou, Baoqiang Ma, Yan Li, Pei Yang, Yingrui Shi, Arjen van der Schaaf, Lisanne V. van Dijk, Johannes A. Langendijk, Nanna M. Sijtsema

**Affiliations:** a Department of Radiation Oncology, University of Groningen, University Medical Center Groningen, Groningen, the Netherlands; b Department of Radiation Oncology, Hunan Cancer Hospital, Xiangya School of Medicine, Central South University, Changsha, Hunan, China; c Image Sciences Institute, University Medical Center Utrecht, Utrecht University, Utrecht, the Netherlands; d Department of Radiation Oncology, China-Japan Friendship Hospital, Beijing, China; e Hunan Institute of Schistosomiasis Control (The Third People's Hospital of Hunan Province), Yueyang, Hunan, China; f Affiliated Nanhua Hospital, University of South China, Hengyang, Hunan, China

**Keywords:** Nasopharyngeal carcinoma, computed tomography, prognosis, treatment outcome, radiomics, deep-learning

## Abstract

**Background and purpose:**

Clinical prognostic models for nasopharyngeal carcinoma (NPC) treated with intensity-modulated radiotherapy (IMRT) with or without chemotherapy remain insufficient to capture tumour heterogeneity. We investigated whether computed tomography (CT)-based signatures add prognostic value for overall survival, progression-free survival, local control and distant control in NPC patients.

**Materials and methods:**

The study population consisted of 1360 patients with stage I–IVa NPC treated with (chemo)IMRT (2013–2017). Radiomic and deep-learning features were analysed with twelve clinical variables. Radiomic models were built using bootstrap resampling feature selection and multivariable Cox regression; deep-learning models used 3D ResNet-18 or DenseNet-121. Models were evaluated on an internal hold-out test set (*n* = 409; training set *n* = 951) with the concordance index and compared against clinical-only reference models. Decision curve analysis was used to assess clinical utility.

**Results:**

Adding radiomic primary tumour features (Neighbouring Gray Tone Difference Matrix - coarseness) improved local control concordance index from 0.51 to 0.60 (*p* = 0.02). A DenseNet-121 combining clinical data with composite primary tumour and lymph node masks achieved the highest distant control (0.68 vs 0.66, *p* = 0.01). For overall survival and progression-free survival, the improvements were not significant. Decision curve analysis demonstrated net benefit of the DenseNet-121 distant control model over treat-all and treat-none strategies at threshold probabilities of 10–25%.

**Conclusions:**

Incorporating CT-based radiomic and deep-learning features into prognostic models significantly improved prediction of local and distant control in NPC, supporting their potential as imaging biomarkers for refined risk stratification.

## Highlights

• Primary tumour radiomics increased local control model C-index from 0.51 to 0.60.
• CT deep learning improved distant control clinical model C-index to 0.68.
• Distant control model demonstrated clinical net benefit at 10–25% risk thresholds.

## Graphical abstract

**Figure:**
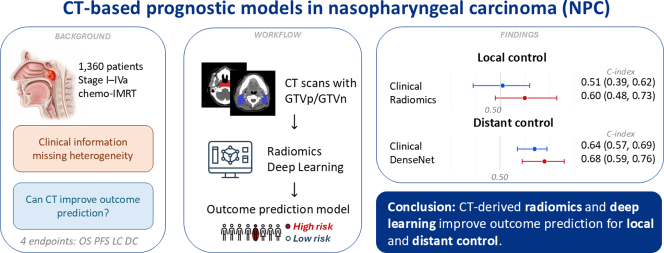

## 1 Introduction

Nasopharyngeal carcinoma (NPC) is a malignancy with distinct geographical and ethnic distributions, prevalent in specific regions especially in Southeast Asia. Although significant improvements in the treatment of NPC have been made since the introduction of intensity-modulated radiotherapy (IMRT) [1], predicting clinical outcomes for NPC patients remains challenging, and patients with similar tumour stages often experience diverse post-treatment outcomes [2], [3]. Current treatment decisions are based on the American Joint Committee on Cancer/Union for International Cancer Control (AJCC/UICC) staging system, supplemented by patient factors such as WHO performance status, age, and Epstein-Barr virus (EBV) DNA levels [4]. However, these methods may not sufficiently quantify the nuances of clinical variables observed in practice and do not reflect the detailed information from medical imaging on tumour heterogeneity [5].

Planning computed tomography (CT) scans provide a valuable foundation for prognostic studies in NPC. They are acquired as part of routine care and already contain clinician-approved contour sets for the primary tumour and involved lymph nodes. In addition, CTs contain high-resolution information on anatomy and spatial context, and their intensity values are relatively stable across institutions. These characteristics make CT widely accessible, cost-effective, and particularly suitable for image biomarker exploration [6]. The predictive utility of CT imaging has been demonstrated in several studies; radiomic models incorporating CT features showed improved predictive performance for overall survival, progression-free survival, and local recurrence [7], [8], [9], [10]. Furthermore, deep-learning approaches, such as using pre-trained convolutional neural networks to identify predictive features from CT images, have demonstrated promise in enhancing predictive performance in identifying treatment responders and non-responders in NPC patients [11].

Despite growing interest in NPC prognostics, most studies address only a single clinical endpoint and rely on either radiomics or deep-learning alone. Furthermore, they often incorporate large feature sets that risk overfitting. To bridge the gap between technical modelling and clinical utility, this study systematically evaluated both signature types derived from routine planning CTs across overall survival, progression-free survival, local and distant control. By comparing each model against clinical-only baselines and employing restrained feature selection, we aimed to deliver highly generalisable and robust predictive tools.

## 2 Materials and methods

### 2.1 Patients

The study consisted of patients with non-metastatic NPC treated with (chemo)IMRT at Hunan Cancer Hospital, China, from January 2013 to December 2017. According to the treatment protocol (Supplementary material A) the prescribed dose was 72 Gy to the primary tumour and 70.4–72 Gy to involved lymph nodes delivered in 32 fractions. Inclusion criteria were as follows: 1) histopathologically confirmed NPC, stage I-IVa according to the 7th AJCC/UICC TNM edition; 2) age 18–75 years, Karnofsky Performance Score ≥ 70, and WHO performance status ≤2; 3) scheduled for curative-intent radiotherapy (with or without chemotherapy); 4) pretreatment contrast-enhanced planning CT of the head-and-neck region available; 5) no previous malignant disease; 6) no previous radiation to the head and neck region. Patients without available CT images, radiotherapy files or delineations were excluded from the study. As this was a retrospective study using anonymised data, the institutional review board of Hunan Cancer Hospital approved this study and waived the requirement for informed consent.

### 2.2 Study endpoints

All patients were followed every three months during the first two years after treatment and every six months for the next 3–5 years. Four endpoints were considered, including overall survival (OS), defined as the time from first day of radiotherapy to any cause of death; progression-free survival (PFS), defined as the time from first day of radiotherapy to first progression (local, regional, or distant) or any cause of death; local control (LC) and distant control (DC) defined as the time from first day of radiotherapy to local recurrence or distant metastasis, respectively. Local recurrence was confirmed by magnetic resonance imaging (MRI) and nasopharyngeal biopsy when clinically suspected. Distant metastasis was identified by MRI, CT, positron emission tomography (PET)/CT, bone scan, and/or abdominal ultrasound based on the suspected site of progression.

### 2.3 Image acquisition and region of interest definition

**Fig. 1:**
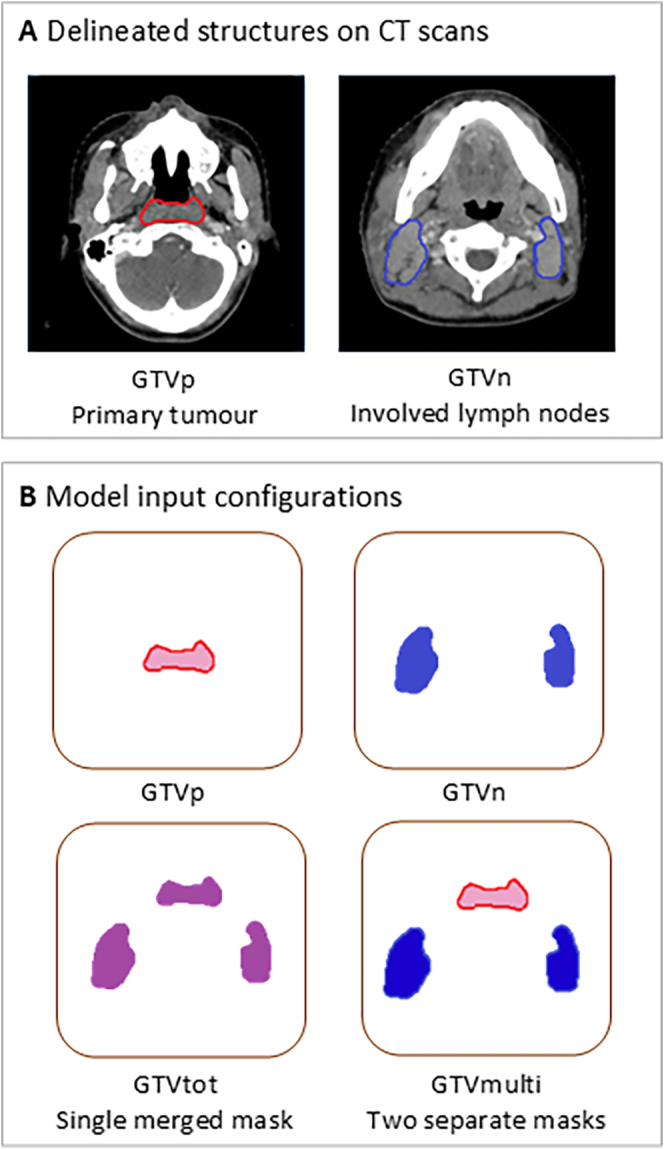
GTV input configurations used for radiomic and deep-learning models. (A) Axial contrast-enhanced planning CT slices showing the delineated GTVs; (B) Four configurations used as model inputs: GTVp alone; GTVn alone; GTVtot combines GTVp and GTVn into a single merged binary mask, used as one input channel. GTVmulti retains GTVp and GTVn as two spatially distinct binary masks, provided as separate input channels. GTV = gross tumour volume.

All CT simulation scans were acquired on a GE LightSpeed RT using 200 mAs and 120 kVp, with a slice thickness of 2.5 mm in the lesion area. To guide the manual segmentation of the regions of interest (ROIs), diagnostic MRI (and PET/CT, when available) were registered with the planning CT images. These delineated ROIs formed the basis of four input configurations used across the models: primary tumour (GTVp), involved lymph nodes (GTVn), GTVtot (where GTVp and GTVn were merged into a single binary mask), and GTVmulti (where GTVp and GTVn were retained as two separate binary masks and provided as distinct input channels). The four configurations are illustrated in Fig. 1.

### 2.4 Clinical data collection and preprocessing

The analysis included twelve variables: sex, age, body-mass index (BMI), WHO performance status, symptom-to-treatment interval, T stage, N stage, AJCC stage, primary-tumour volume, smoking pack-years, plasma EBV-DNA level, and chemotherapy regimen (induction, concurrent, adjuvant chemotherapy, or a combination). Patients were staged according to the 7th edition AJCC/UICC staging system, as the treatment occurred before December of 2017. EBV-DNA levels were categorised into two groups; T category was recoded as a binary factor (T1-T3 = 0; T4a = 1), and N category likewise (N0-N2 = 0; N3 = 1), based on survival trends seen in an earlier study of clinical models for this cohort [12]. The primary-tumour volume was derived from GTVp. The clinical-only model was refit on the current cohort to ensure a fair comparison with the imaging-derived models.

### 2.5 Data split and imputation

**Fig. 2:**
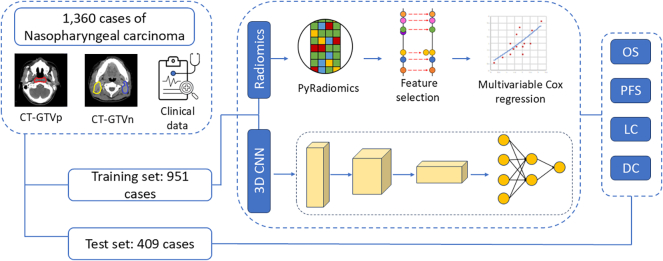
Study workflow overview. A total of 1360 patients with nasopharyngeal carcinoma were included and randomly split into training (951 patients) and internal test (409 patients) sets. Radiomic features were extracted using PyRadiomics, followed by feature selection and multivariable Cox regression modelling. In parallel, deep-learning models were developed using three-dimensional convolutional neural networks (3D CNNs). Both radiomic and deep-learning models were evaluated in the test set for predicting four clinical endpoints: overall survival (OS), progression-free survival (PFS), local control (LC), and distant control (DC).

The overall study workflow for the radiomics- and deep-learning-based models is summarised in Fig. 2. The dataset was split into a training (70%) and a testing (30%) cohort using stratified sampling based on event occurrence and treatment initiation date to balance event rates and minimise systematic differences in treatment protocols over time. Missing covariate values (pack-years and EBV-DNA) were imputed using multiple imputation (m = 10) in both cohorts independently. Detailed procedures regarding cohort comparison and the pooling of imputation models are provided in Supplementary Material B.

### 2.6 Radiomic feature extraction and model development

A total of 107 radiomic features were extracted from the CT images using PyRadiomics (v 3.1.0; Python 3.7) [13], code available at https://github.com/guanzhi-ops/radiomic-cox-analysis. Because all CT scans were acquired on a single scanner using a standardised protocol, cross-scanner harmonisation was not required. To prevent selection bias from excluding node-negative cases (*n* = 62), missing GTVn data were imputed to reflect the absence of nodal disease (details in Supplementary Material B). Feature selection was conducted by preselecting features based on Pearson correlation (<0.8) and univariable association, followed by forward selection with bootstrapping (1000 iterations). This was performed under three settings: (1) Radiomic features selected while mandatorily including previously identified significant clinical parameters (Forced); (2) Unbiased selection from both clinical and radiomic features simultaneously (Unbiased); and (3) Radiomic features only (Rad-only). Multivariable Cox regression models were subsequently developed for each outcome.

### 2.7 Deep-learning model development

CT images and GTV masks were cropped to a 192 × 192 × 192 mm^3^ bounding box centred around the GTV, resampled to a 2 × 2 × 2 mm^3^ resolution, truncated (−200 to 200 HU), and z-score normalised. Two three-dimensional convolutional networks (ResNet-18 and DenseNet-121) were evaluated. Model input options included GTVp, GTVtot, and GTVmulti; input GTVn set to zero for node-negative cases. Clinical variables were incorporated through fully connected layers, and a clinical-only network served as a reference. Models were trained using event-stratified five-fold cross validation. The test-set risk scores were obtained by averaging over the five model predictions. Comprehensive details regarding model optimisation, loss functions, software/hardware configurations, and data augmentation are described in Supplementary Material B.

### 2.8 Model validation

Clinical-only Cox and deep-learning models were developed as reference models. For both radiomic and deep-learning models, discrimination performance was assessed using Harrell's concordance index (C-index) with 95% confidence intervals (95% CI). Four endpoints were analysed as independent pre-specified comparisons; no correction for multiple comparisons was applied. The C-index of each CT-based model was compared with the clinical baseline using the likelihood-ratio test (LRT). Given the modest censoring rate, calibration at 5 years was assessed by dichotomising predicted probabilities into binary outcomes, using the Hosmer–Lemeshow χ^2^ test and calibration plots for visual assessment. For the LC endpoint with limited number of events, Uno's C-index (inverse probability of censoring weighting adjusted) and time-dependent Brier scores were additionally computed to assess discrimination under heavy censoring and calibration over time, respectively.

### 2.9 Clinical utility and model interpretability

To assess the clinical utility of the CT-based models, risk stratification was evaluated by dichotomising predicted risk scores at the mean into low- and high-risk groups and comparing survival outcomes using Kaplan-Meier analysis. Decision curve analysis (DCA) was performed to estimate the net clinical benefit across a range of clinically plausible threshold probabilities at 60 months of follow-up, benchmarked against the treat-all and treat-none reference strategies. Saliency maps were generated using Grad-CAM applied to the final convolutional layer of the DL-based model to visually assess which image regions most influenced model predictions.

## 3 Results

A total of 1360 patients met the inclusion criteria of the study and were included in the analysis (Fig. S1). There were no significant differences in patient characteristics, event rates, or missing data between the training and test sets (Table 1). All models demonstrated good calibration (Fig. S3).

**Table 1:** Baseline patient characteristics and event rates in the training and test cohorts. Values are presented as mean ± SD for continuous variables and as *n* (%) for categorical variables. *P* values were calculated using χ^2^ tests for categorical variables and *t*-tests (or Mann–Whitney *U* tests) for continuous variables.

Characteristic	Training set (*n = 951*)	Test set (*n = 409)*	*P* value
Age, years	48.1 ± 10.1	47.8 ± 10.0	0.58

Sex			0.85
Male	691 (72.7%)	300 (73.3%)	
Female	260 (27.3%)	109 (26.7%)	
BMI, kg/m^2^	23.3 ± 3.2	23.4 ± 3.4	0.56

T-stage			0.79
T1–3	699 (73.5%)	297 (72.6%)	
T4a	252 (26.5%)	112 (27.4%)	

N-stage			0.95
N0–2	699 (73.5%)	302 (73.8%)	
N3	252 (26.5%)	107 (26.2%)	
Symptom-to-treatment interval^a^, months	7.1 ± 6.4	6.9 ± 7.3	0.68

WHO performance status			0.32
0	366 (38.5%)	170 (41.6%)	
1–2	585 (61.5%)	239 (58.4%)	

EBV status^#^			0.51
Negative	441 (46.4%)	181 (44.3%)	
Positive	510 (53.6%)	228 (55.7%)	
Primary volume^b^, cm^3^	44.9 ± 37.3	46.7 ± 38.3	0.43
Pack-years of smoking^#^	14.8 ± 21.4	13.3 ± 18.2	0.21

Events during follow-up			
Mortality	138 (14.5%)	60 (14.7%)	>0.99
Progression^c^	215 (22.6%)	94 (23.0%)	0.94
Local failure	50 (5.3%)	21 (5.1%)	>0.99
Distant failure	96 (10.1%)	42 (10.3%)	>0.99

Abbreviations: BMI = body mass index; EBV = Epstein-Barr virus; SD = standard deviation; WHO = World Health Organization.

Notes. ^a^Defined as the time from first symptom to treatment start. ^b^Derived from the gross tumour volume (GTV) used for radiotherapy target delineation. ^c^Progression events include local, regional, or distant failure and death from any cause. ^#^EBV-DNA status and pack-years of smoking had 53.3% and 4.0% missing data, respectively, and were imputed using multiple imputation performed independently in the training and test sets. EBV status was dichotomised using a threshold defined in the training set.

Incorporating radiomic features for OS, PFS (either forced or unbiased selection methods) resulted in a small improvement in model performance with respect to the clinical Cox model (Table 2); however, this improvement was not significant. The LC model combining clinical and radiomic parameters demonstrated significant improvement in model fit over the clinical-only Cox model (LRT *p* = 0.02) and a better discrimination. Uno's C-index confirmed the discrimination gain of the radiomic model over the clinical model (ΔC = 0.09; Table S5). Brier scores were low at both time points (Table S5). The best performing radiomic models included the primary tumour volume and the radiomic texture feature coarseness determined from the Neighbouring Gray Tone Difference Matrix of GTVp (GTVp-NGTDM-Coarseness) as input parameters (Table S1.1). Representative cases illustrated the added predictive value of the radiomic feature GTVp-NGTDM-Coarseness (Fig. 4). For DC, the radiomic-only model including a shape feature of GTVn (least axis length) and a texture feature of GTVp (Zone Percentage of the Gray Level Size Zone Matrix) was comparable to the clinical Cox model (Table 2). Detailed stepwise forward selection of the final model was presented in Table S3. Univariable analysis results, preselected features, and bootstrap forward selection results for each endpoint are provided in Supplementary Material C.

**Table 2:** Comparison of the performance of the final models in the test set. For each endpoint (OS, PFS, LC, DC), the table reports the C-index (95% CI) for: (i) a Cox model with clinical variables only; (ii) the best Cox model including clinical variables and CT radiomic features as input; (iii) a deep-learning model using clinical variables only; (iv) the best CT-based deep-learning model including clinical variables. The final column details the model type and the input configuration of the best-performing model for each endpoint.

Endpoint	Cox model: Clinical only	Cox model: Clinical + CT radiomic features	Deep-learning model: Clinical only	Deep-learning model: Clinical + CT	Best model input
OS	0.68 (0.62, 0.75)	0.69 (0.62, 0.76)	0.70 (0.62, 0.75)	0.69 (0.63, 0.75)	DL (clinical)
PFS	0.63 (0.57, 0.69)	0.64 (0.58, 0.69)	0.64 (0.58, 0.70)	0.66 (0.60, 0.71)	DL (clinical + GTV_multi)_
LC	0.51 (0.39, 0.62)	0.60 (0.48, 0.73)^⁎^	0.55 (0.40, 0.64)	0.54 (0.41, 0.66)	Cox (clinical + GTV_p_)
DC	0.64 (0.57, 0.69)	0.64 (0.56, 0.72)	0.66 (0.56, 0.73)	0.68 (0.59, 0.76)^#^	DL (clinical + GTV_tot_)

^⁎^indicates significant improvement over the clinical only Cox model, and ^#^ indicates significant improvement over the deep-learning clinical model. Bold indicates the best-performing model for each endpoint.

Abbreviations: GTV_p_ = GTV primary tumour features, GTV_multi_ = considering GTV of primary tumour and GTV of lymph nodes as two separate structures as two channels of the model input, GTV_tot_ = considering GTV of primary tumour and GTV of lymph nodes as one whole structure.

Among all evaluated deep-learning model types and input combinations, the deep-learning models using only clinical variables achieved the highest C-index values for OS (Table 2). For PFS prediction, the best-performing model was the CT-based ResNet-18 model incorporating clinical variables in combination with GTVmulti as input. For DC prediction, the highest C-index was achieved by the CT-based DenseNet-121 models using clinical variables and GTVtot as input, which performed significantly better than the clinical deep-learning model (*p* = 0.01; Table 2). Full results across all radiomic and deep-learning configurations are provided in Tables S1.1 and S1.2.

**Fig. 3:**
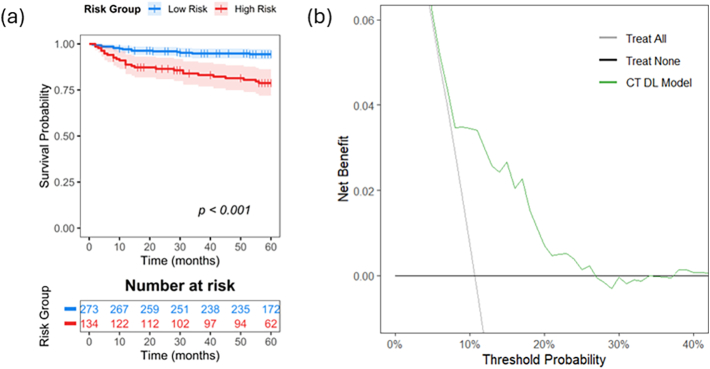
Risk stratification and net clinical benefit of the CT-based deep-learning model for distant control. (a) Kaplan-Meier survival curves with 95% confidence intervals for distant control at 60 months, stratified by predicted risk group, in the test set. (b) Decision curve analysis of the CT-based deep-learning model for distant control at 60 months in the test set, compared with treat-all and treat-none reference strategies.

For OS, PFS and DC, both the CT-based deep-learning model and the CT-based radiomic model achieved statistically significant separation between risk groups (log-rank *p* < 0.01); risk group separation was not statistically significant for either model for LC (*p* = 0.53 and *p* = 0.14, respectively) (Fig. S4). Decision curve analysis for the CT-based deep-learning model for DC demonstrated net clinical benefit over both the treat-all and treat-none reference strategies at threshold probabilities between approximately 10% and 25% (Fig. 3; Fig. S5). DCA for LC was not performed owing to the low number of events in the test set. Saliency maps of two example patients demonstrated that the DL models mainly used features around the primary tumour and involved lymph node regions for the prediction (Fig. S6).

## 4 Discussion

This study demonstrated that routine planning CT imaging carried prognostic information beyond standard clinical variables for NPC patients treated with (chemo)IMRT. The poor performance of the refitted clinical-only model for LC suggested that standard clinical variables offer limited discriminative value for this endpoint, pointing to the need for imaging-derived features. Radiomic features significantly improved LC prediction, while deep-learning models enhanced DC prediction. No significant improvement was observed for OS or PFS.

**Fig. 4:**
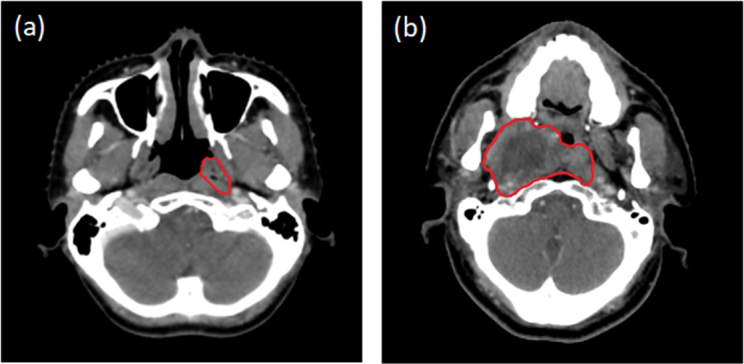
Illustrative examples of local recurrence risk stratification using radiomic features. (a) Predicted high risk of local recurrence with small volume (2.7 cm^3^) and high coarseness (4.68), the event occurred. (b) Predicted low risk of local recurrence with large volume (100.3 cm^3^) and low coarseness (−0.74), no event occurred.

The added value of CT imaging for LC was driven by two radiomic features: GTVp volume and GTVp NGTDM-coarseness. NGTDM-coarseness quantifies the rate of gray level change between neighbouring voxels; higher coarseness values reflect a coarser texture and were associated with a higher risk of local recurrence, consistent with a more aggressive tumour phenotype [14]. Together, these features suggested that both shape and texture characteristics of the GTVp are more predictive for LC than clinical factors alone. Adding NGTDM-coarseness to GTVp volume improved predictive power, particularly for tumours of comparable size, as illustrated in Fig. 4.

For DC prediction, the deep-learning approach achieved significantly better performance than the clinical Cox model. When trained on GTVtot along with clinical variables, it reached the highest performance across all configurations. This result was in agreement with earlier findings in oropharyngeal cancer, where incorporating both primary tumour and nodal information likewise improved DC prediction [15]. A similar pattern was observed in the radiomic model. In our cohort, nodal features were stronger predictors in univariable analysis (Fig. S2) and were the most frequently selected in bootstrap resampling forward selection (Table S2.1, *DC GTVmulti*). This finding aligned with the AJCC staging, which emphasises nodal extent in the assessment of distant metastasis risk [4].

Another key observation was that radiomic models performed better for LC prediction, whereas the DL model was superior for DC. Several reasons may explain this difference. First, the lower event rate for LC compared to DC may have limited the performance of the deep-learning approach, as deep-learning models typically struggle with imbalanced datasets [16]. Second, local recurrence depends mostly on tumour-specific characteristics such as local heterogeneity, explicitly quantified by radiomic metrics like NGTDM-coarseness [14]. In contrast, distant metastasis involves more complex biological processes, such as lymphatic spread and vascular invasion, which can potentially be captured better by deep-learning models by evaluating patterns across multiple regions [17].

For OS and PFS, the addition of CT features did not significantly improve the clinical model. Clinical variables appeared to capture the relevant sources of prognostic variation for these endpoints, leaving limited residual information to be extracted from CT.

The strengths of this study were the large single-centre cohort acquired with a uniform CT protocol, and the systematic comparison of both radiomic and deep-learning signatures across four endpoints against clinical baselines. The use of restrained bootstrap feature selection further limited the risk of overfitting. Several limitations of our study should be noted. First, for LC, although the C-index improved, the absolute value remained low. The low event rate limited the power of risk group analyses and made decision curve analysis unreliable. Performance metrics for this endpoint are also sensitive to statistical noise, making evaluation of high-dimensional deep-learning models difficult. Larger datasets with more LC events are needed to develop and evaluate prognostic models for this endpoint reliably. Second, some clinically important variables, such as EBV status and chemotherapy information, were not selected in the final model [18]. This could be related to the fact that 53.3% of the EBV DNA values were imputed, which could have influenced its prognostic value and chemotherapy was recorded only as general treatment type without regimen or dose. Third, this study lacks external validation; future work should prioritise validating these models in independent cohorts with different imaging protocols and diverse patient populations before clinical implementation can be considered.

Additionally, prospective validation appears practical, given that the required inputs can be easily extracted from standard clinical radiotherapy workflows. After successful validation, these models could be used to guide more personalised treatment approaches, which have the potential to improve treatment outcomes for NPC patients.

In summary, CT-based prognostic models for nasopharyngeal carcinoma, integrating routine planning CT with clinical factors were developed and compared across overall survival, progression-free survival, local control, and distant control. Radiomic features from the primary tumour improved LC prediction, while a deep-learning model incorporating CT information from the primary tumour and involved lymph nodes enhanced DC prediction. These findings highlighted the potential of CT imaging signatures to significantly refine prognostic accuracy over clinical baselines, supporting more personalised and effective treatment planning for NPC patients.

## CRediT authorship contribution statement

**Guanzhi Zhou:** Writing – original draft, Visualization, Investigation, Funding acquisition, Formal analysis. **Baoqiang Ma:** Writing – original draft, Validation, Software, Formal analysis. **Yan Li:** Writing – review & editing, Software, Formal analysis, Data curation. **Pei Yang:** Writing – review & editing, Supervision, Resources, Project administration, Methodology, Funding acquisition, Conceptualization. **Yingrui Shi:** Writing – review & editing, Supervision, Resources, Investigation. **Arjen van der Schaaf:** Writing – review & editing, Methodology. **Lisanne V. van Dijk:** Writing – review & editing, Supervision, Methodology. **Johannes A. Langendijk:** Writing – review & editing, Supervision, Resources, Funding acquisition, Conceptualization. **Nanna M. Sijtsema:** Writing – review & editing, Supervision, Resources, Methodology, Conceptualization.

## Funding

This work was funded by the Chinese Scholarship Council (CSC).

## Declaration of competing interest

The authors declare the following financial interests/personal relationships which may be considered as potential competing interests: Prof. Langendijk was a member of the Global Advisory Board of IBA (honorarium paid to the UMCG Research BV) and the RayCare International Advisory Board of RaySearch. The department of Radiation Oncology of UMCG has research agreements with IBA, RaySearch, Siemens, Elekta, Mirada, and Leoni. All other authors declare no competing interests.

## Appendix A Supplementary data

Supplementary material containing the treatment protocol, extended modelling methodology, detailed results of the radiomics models, and supplementary figures and tables.
