# Supplementary material for: Integrating computed tomography image features improves clinical prediction models for outcomes in nasopharyngeal carcinoma patients treated with (chemo)radiation
Source: Phys Imaging Radiat Oncol. 2026 Jul 13;40:101040. doi: 10.1016/j.phro.2026.101040 (PMC13416826; doi:10.1016/j.phro.2026.101040)
Supplement: Supplementary file 1 — Supplementary material containing the treatment protocol, extended modelling methodology, detailed results of the radiomics models, and supplementary figures and tables. [file mmc1.pdf]

Supplementary materials

Table of contents:

Supplementary materials A. Treatment protocol

Supplementary materials B. Extended Modelling Methodology

Supplementary materials C. Detailed results of radiomics models

Supplementary Figures and tables

Abbreviations

Supplementary materials A. Treatment protocol

A.1 Radiotherapy Protocol

A.1(a) Target Volume Definitions

- **Gross Tumour Volume (GTV)**
  - **GTVp:** The gross tumour volume of the primary nasopharyngeal carcinoma, defined based on clinical examination, endoscopy, and imaging.
  - **GTVn:** The gross tumour volume of cervical lymph node metastases, delineated from clinical and imaging findings.
- **Clinical Target Volume (CTV):**
  - **CTV1**: Includes the GTV with an expansion of 0.5–1.0 cm anteriorly, superiorly, inferiorly, and laterally, and an expansion of 0.3–0.5 cm posteriorly (with adjustments based on tumour involvement and proximity to critical structures such as the spinal cord or brainstem).
  - **CTVn:** For metastatic cervical lymph nodes, defined by expanding the GTVn along the fat plane by 0.5–1.0 cm.
  - **CTV2**: An extension of CTV1 by an additional 0.5–1.0 cm (anteriorly, superiorly, laterally) and 0.3–0.5 cm posteriorly; it also includes CTVn, the corresponding lymphatic drainage regions, and prophylactically treated nodal areas.
- **Planning Target Volume (PTV):**
  - The PTV is generated by expanding each target volume by 3–5 mm (3 mm if image guidance is used; 5 mm if not).

A.1(b) Fractionation Schedules and Prescription Doses

- **Primary Tumour and Lymph Nodes:**
  - GTVp: 2.25 Gy per fraction × 32 fractions = 72 Gy.
  - GTVn: 2.20-2.25 Gy per fraction × 32 fractions = 70.4-72 Gy.
- **Planning Target Volumes:**
  - PTVp: 2.2 Gy per fraction × 32 fractions = 70.4 Gy.
  - PCTV1 and PTVn: 1.9 Gy per fraction × 32 fractions = 60.8 Gy.
  - PCTV2: 1.72 Gy per fraction × 32 fractions = 55 Gy.

A.1(c) Additional Note: An MRI evaluation is performed after 18–20 fractions. In patients with clearly residual tumour and good overall condition (i.e., absence of high-risk factors such as malnutrition), the single fraction dose for GTVp may be increased to 2.3 Gy, as appropriate.

A.2 Chemotherapy Information

A.2(a) Chemotherapy Regimens

- Induction Chemotherapy (ICT):
  1. Patients received 1-2 cycles of chemotherapy before radiotherapy.
  2. Common regimens included TP (Paclitaxel and Nedaplatin) and DP (Docetaxel and Cisplatin).
- Concurrent Chemoradiotherapy (CCRT):
  1. CCRT combined chemotherapy with radiotherapy.
  2. The most common regimen was TP (Paclitaxel and Nedaplatin).

- Adjuvant Chemotherapy (ACT):
    1. ACT was given after radiotherapy to reduce recurrence risk.

A.2(b) Eligibility Criteria

Eligibility for chemotherapy was based on the following:

- Tumour Staging
    - Stage I: Most received CCRT due to limited tumour burden, with a few receiving ACT for high-risk features.
    - Stage II: Many underwent ICT followed by CCRT for locally advanced tumours. Some received ACT for high recurrence risk.
    - Stage III: Most received ICT and CCRT. ACT was used for residual disease post-radiotherapy.
    - Stage IVa: Most received ICT for extensive disease, followed by CCRT. ACT was used selectively for high-risk cases.
- Performance Status: Adequate status to tolerate treatment.
- Clinical Assessment: Individualised evaluations ensured optimal treatment.

Supplementary materials B. Extended Modelling Methodology

B.1 Data Split and Imputation Protocols

Following the train-test split, patient characteristics between the training and test cohorts were statistically compared to confirm the absence of significant differences. Missing clinical covariates were imputed using the mice package in R (v 4.2.1), following the procedure described in our previous clinical-variable model study [1]. Upon completion of multivariable modelling, Rubin's rule [2] was applied to pool coefficients, hazard ratios, and p-values of the model parameters across the ten imputation sets.

B.2 Radiomic Feature Imputation for Node-Negative Cases

To ensure the inclusion of all 1,360 patients, radiomic features for the 62 node-negative cases were imputed to reflect the most favourable nodal scenario. Specifically, radiomic features demonstrating a positive univariable prognostic coefficient were assigned the training-set's minimum observed value, while features with a negative coefficient were assigned the training-set's maximum observed value. This rule was used rather than a single fixed constant (e.g. zero), because a common constant cannot consistently represent nodal absence across radiomic features. For size and volume features, zero naturally encodes an absent node; for other features, however, their value-risk relationship is feature-dependent (a high value can indicate either higher or lower risk depending on the feature), and some features are negative throughout the cohort. We therefore assigned each feature to its training-set minimum or maximum according to the sign of its univariable coefficient, to reflect the least nodal contribution to risk. The test set was not used in this procedure; no test set outcome information entered the imputation.

B.3 Deep Learning Optimisation and Hardware

Deep learning experiments were implemented in PyTorch (v 1.12.1) and MONAI (v 1.1.0) and executed on an NVIDIA Tesla A100 GPU. The 3D ResNet-18 and DenseNet-121 architectures utilised their default MONAI configurations. A Cox partial likelihood loss was used to optimise the survival prediction models, following the training procedure of our previous head and neck workflow [15]. Model optimisation employed stochastic gradient descent (SGD) with a momentum of 0.9 and an initial learning rate of 0.0002. Early stopping with a patience of 25 epochs was applied to retain the model checkpoint that achieved the best validation performance.

B.4 Data Augmentation and Sampling

During training, data augmentation was applied to improve model generalisation. Transformations included random flipping along all three axes (probability = 0.5), random affine transformations (probability = 0.5), and random elastic deformations (probability = 0.2). Furthermore, to mitigate event imbalance within the dataset, oversampling was utilised during the training phase to maintain an equal representation of patients experiencing events and censored cases.

Supplementary materials C. Detailed results of radiomics models

The forest plots (**Figure S2**) summarise the results of the univariable analysis of clinical and radiomic predictors: Hazard Ratios (HR) and 95% confidence intervals of the predictors with the different endpoints are shown. **Table S2** summarises feature selection outcomes across 1000 bootstrap iterations. For each endpoint there are nine tables that report the selection frequency for the selected features in different scenarios: *rad-only*, *forced* selection and *unbiased* selection. Separate tables are shown for features from *GTVp only, GTVn only*, and combined GTV (*GTV multi*). The best-performing scenario for each endpoint is marked in the table. **Table S3** illustrates the stepwise forward selection process, showing how coefficients and the corresponding C-index changed at each step for the final model. Finally, **Table S4** summarises the final parameters, coefficients, and hazard ratios of the radiomics models. The key takeaway results for each endpoint are as follows:

C.1 Overall survival

Univariable Analyses and Feature Preselection

T stage (HR = 2.4, $p < 0.001$) and N stage (HR = 1.9, $p = 0.003$) were the strongest clinical predictors of OS (Fig S2). Among radiomic features, GTVn-Shape metrics demonstrated prognostic relevance, with Least Axis Length (LAL) (HR = 1.8, $p = 0.01$) and Flatness (HR = 1.6, $p = 0.02$) achieving statistical significance. Following correlation-based preselection (threshold: $r > 0.8$), 43 features (9 clinical, 34 radiomic) were retained for OS modelling.

Bootstrapped Forward Selection Results

The bootstrapping frequency table is shown in Table S2. In the **forced** setting, with *Age, T, N, EBV DNA* forced in the model, nodal shape features were consistently prioritised, with *GTVn-shape Least Axis Length (LAL)* and *GTVn-shape Flatness* selected in 65% and 46% of iterations, respectively. Additionally, the *GTVn-shape Maximum 2D Diameter Row (MDR)* emerged as a stable first-selection candidate (32%), indicating its independent prognostic value.

In the **unbiased** setting, *age* was the dominant feature, selected in nearly all iterations (99%). The role of *T stage* varied depending on the inclusion of primary tumour radiomic features—when only nodal radiomics were considered, *T stage* had a higher selection frequency (48%), but when primary tumour features (e.g., *Gray Level Non-Uniformity, Strength*) were introduced, *T stage* was selected far less often (22%). Notably, neither *T stage* nor primary tumour features were selected as frequently as nodal radiomic features.

Final model performance

In both forced and unbiased settings, models based on features from different sites yielded identical discrimination (C-index = 0.69). The same discrimination was retained when the clinical parameters T stage and EBV DNA were excluded (Table S3). Key predictors for OS included *Age* (HR = 27.4, 95% CI = 11.3, 66.6, $p < 0.01$), *N stage* (ΔC-index = +0.04, $p < 0.01$), and *GTVn-shape MDR* (ΔC-index = +0.02, $p < 0.01$). The performance was poor for radiomics only models, indicating the necessity of integrating clinical variables in this endpoint.

C.2 Progression-Free Survival

Univariable Analyses and Feature Preselection

N stage (HR = 1.7, p < 0.01), EBV DNA (HR = 1.7, p < 0.01), and *T stage* (HR = 1.6, p < 0.01) were dominant clinical predictors. Radiomics highlighted GTVn-shape LAL (HR = 1.3, p < 0.01) and GTVp-shape-LAL (HR = 1.3, p < 0.01) (Fig S2). After correlation filtering, 43 features (9 clinical, 34 radiomic) were selected as candidate features.

Bootstrapped Forward Selection Results

In the **forced setting**, with *Age, volume, N, pack-years* forced in the model, both nodal and primary tumour radiomic features contributed to the model (Table S2). Specifically, *GTVp-NGTDM Busyness* was the most frequently selected feature (53%), followed by *GTVn-shape Flatness* (39%). In the **unbiased setting**, selection was led by clinical variables, with *age* and *pack-years* appearing consistently, which are also in the referenced clinical model. While primary *tumour volume* and *GTVp-NGTDM Busyness* initially showed some importance, their predictive contributions diminished after adjusting for other features, whereas GTVn-shape LAL and *GTVp-shape LAL* remained stable selections.

Final model performance

The forced and unbiased models performed similarly, achieving a C-index of 0.66, modestly improving upon the clinical model (C-index = 0.64) (Table S3). Notably, primary tumour volume was absent in the unbiased model, suggesting its effect was a confounder of GTVp-NGTDM Busyness for PFS prediction.

C.3 Local control

Univariable Analysis and Feature Preselection

From the univariable analysis (using a significance threshold of p<0.1) for local control, *primary tumour volume, GTVp-NGTDM-Coarseness*, and *GTVp-first order-Maximum* were identified as potentially relevant predictors of LC (Figure S2). The preselection was performed without considering the univariable significance, resulting in 46 features entered in the forward selection (10 clinical, 36 radiomic).

Bootstrapping Forward Selection

The frequencies at which preselected features were chosen as the first, second, or third predictor are summarised in Table S2.

Rad-only: When only including radiomics features in the selection, *GTVp-NGTDM-Coarseness* was the most frequently selected predictor (42% selection frequency). Shape features *GTVp-LAL* followed (frequency: 32%).

Forced: When forcing the referenced clinical model feature (primary tumour volume) in the model, *GTVp-NGTDM-Coarseness* remained a top-ranked feature, indicating its added predictive value to tumour volume.

Unbiased: In the unbiased selection procedure with clinical and radiomics features, both *GTVp-NGTDM-Coarseness* (frequency: 36%) and *tumour volume* (frequency: 20%) were consistently chosen, confirming that including these two features is beneficial for LC in all scenarios.

Table S2 provides the specific frequency for each setting. It is worth mentioning that lymph node features were not selected in any of the LC models with separate features for primary tumours and lymph nodes (LC multi).

Stepwise Forward Selection Result and Final Model

Based on the feature ranking results from the bootstrapped forward selection, stepwise forward selection was performed on the training set to build the final predictive model. Table S3 summarises model improvements at each selection step. *Primary tumour volume* was included in step one, with a modest association with local control (HR = 1.25, 95% CI: 0.97–1.62; p = 0.08) and yielded a low C-index in the test set of 0.51. In Step two, the addition of *GTVp-NGTDM-Coarseness* not only emerged as a significant independent predictor (HR = 1.24, 95% CI: 1.11–1.39; p < 0.01) but also strengthened the association of volume (HR = 1.35, 95% CI: 1.08–1.68; p < 0.01). The final C-index in the training set after adding the two radiomics features was 0.60.

Final Model Performance

When tested in the independent test set, the combined clinical-radiomic model improved the prediction of LC (C-index of 0.60, 95% CI: 0.48–0.73) significantly outperforming the clinical-only model (C-index: 0.51, 95% CI: 0.39–0.62; p<0.05).

C.4 Distant control

Univariable Analysis and Feature Preselection

As shown in Fig S2, *EBV DNA* (HR = 3.1, p < 0.01) and *N stage* (HR = 1.9, p<0.01) emerged as the strongest predictors of DC, followed by radiomic features from GTVn and GTVp regions including *GTVn-shape-LAL* (HR = 1.4, p = 0.03) and *GTVp-GLDM Large Dependence Low Gray-Level Emphasis (LDE)* (HR = 1.3, p = 0.04). Following preselection (r > 0.8), 45 features (9 clinical, 34 radiomic) were retained for modelling.

Bootstrapped Forward Selection

In the forced setting, with *EBV DNA, pack-years,* and *volume* forced in the model, nodal radiomics were predominantly selected, with *GTVn-glszm Gray-Level Non-Uniformity (GLNU)* (32% frequency), which is not reflected in the univariable analysis and *GTVn-shape LAL* (31%) as top features (Table S2). Frequently selected primary tumour features (e.g., *GLSZM Gray Level Variance*) indicated that combining nodal shape and primary tumour texture features could enhance predictive accuracy.

In the **radiomics-only** setting, the *GTVn-shape-LAL* had the highest frequency of being selected as the first variable (49%). Among primary tumour features, *GTVp-GLSZM-ZonePercentage* has the highest selection frequency when added to the model, indicating its added value to the existing nodal variable. In the **unbiased** setting, clinical variable *EBV DNA* led selection (94% frequency). Radiomics follows closely, with *GTVn-shape LAL* (34%) and *GTVn-glszm GLNU* (21%) as top nodal candidates.

Final Model Performance

The forced model was excluded because the addition of radiomics features yielded a non-significant performance improvement and failed to shift the clinical variable coefficients by more than 10%. The radiomics-only model achieved a C-index of 0.64 (0.56, 0.72) in the test set, comparable to the clinical benchmark model, whose C-index was 0.64 (0.57, 0.69). Key predictors included *GTVn-shape-LeastAxisLength* (HR = 1.4, 95% CI = 1.2–1.7, p = 0.01) and

*GTVp-GLSZM-ZonePercentage (*HR = 0.7, 95% CI = 0.6-0.9, p = 0.01*)*

Reference:

[1] Zhou G, Sijtsema NM, Li Y, Zhai T, Xi L, Li J, et al. Prediction of treatment outcome in nasopharyngeal carcinoma patients. BMC Cancer 2026;26:585. https://doi.org/10.1186/s12885-026-15570-w.

[2] Rubin DB. Multiple imputation for nonresponse in surveys. New York: John Wiley & Sons; 1987.

Supplementary Figures and tables

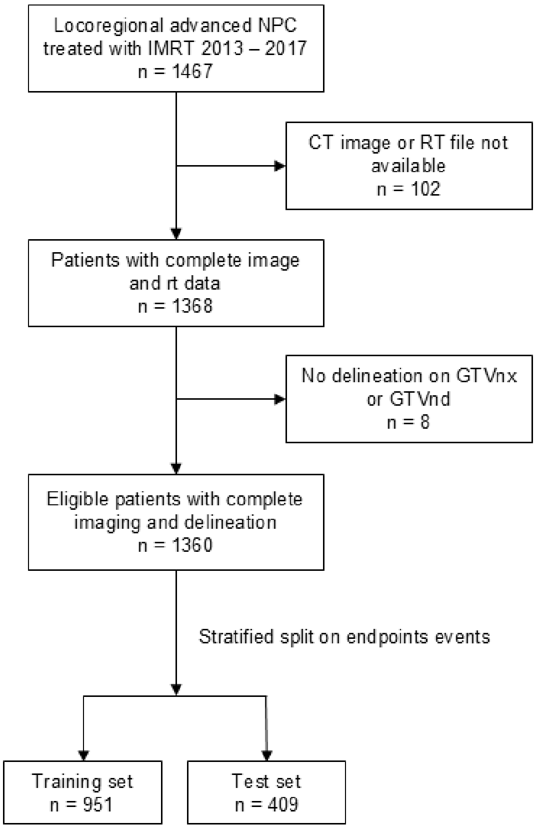

Figure S1. Flowchart of patient selection for training and test cohorts, including screening, eligibility, exclusions, and final sample sizes with reasons for exclusion.

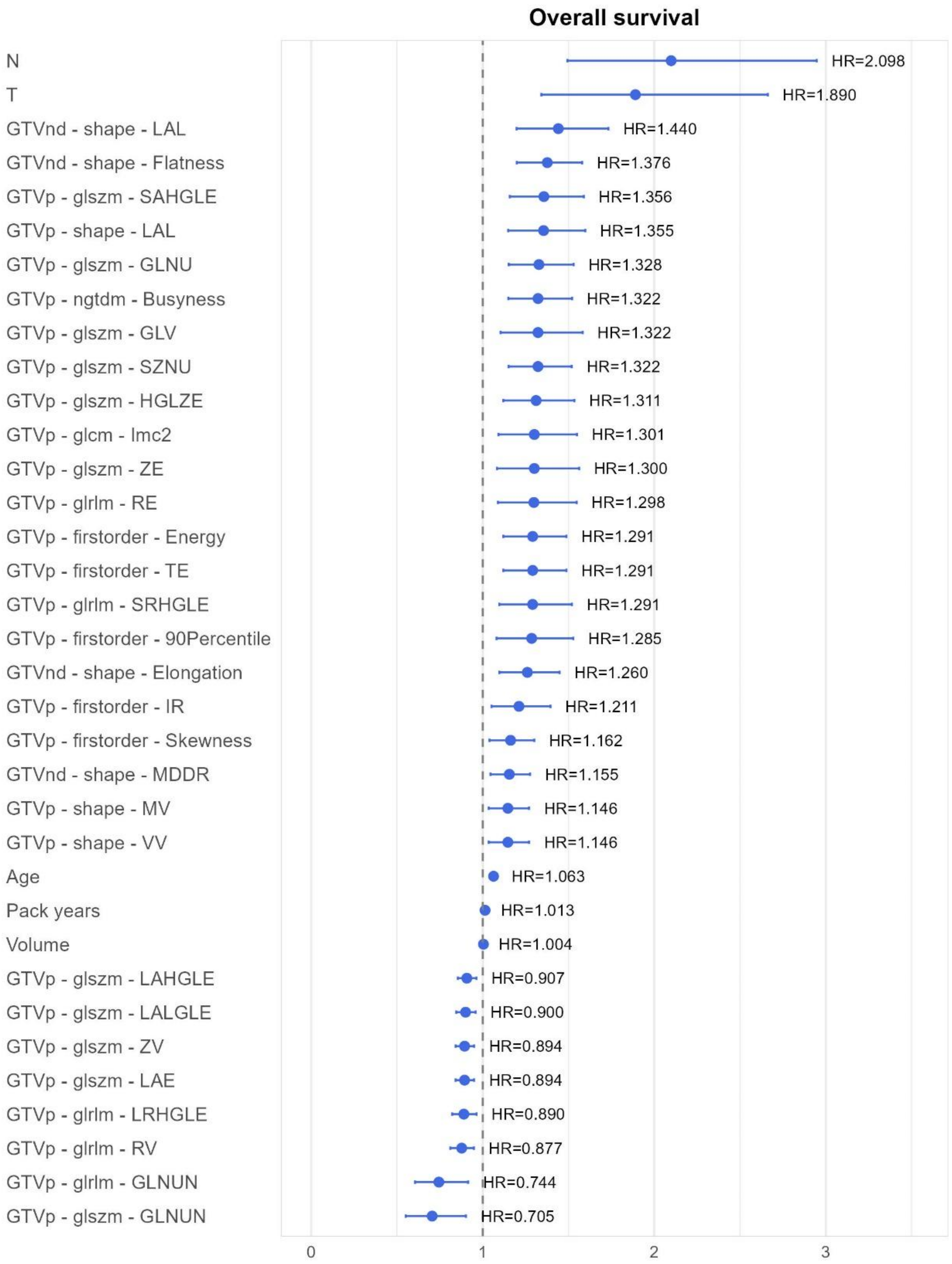
Overall survival
N
T
GTVnd - shape - LAL
GTVnd - shape - Flatness
GTVp - glszm - SAHGLE
GTVp - shape - LAL
GTVp - glszm - GLNU
GTVp - ngtdm - Busyness
GTVp - glszm - GLV
GTVp - glszm - SZNU
GTVp - glszm - HGLZE
GTVp - glcm - Imc2
GTVp - glszm - ZE
GTVp - glrlm - RE
GTVp - firstorder - Energy
GTVp - firstorder - TE
GTVp - glrlm - SRHGLE
GTVp - firstorder - 90Percentile
GTVnd - shape - Elongation
GTVp - firstorder - IR
GTVp - firstorder - Skewness
GTVnd - shape - MDDR
GTVp - shape - MV
GTVp - shape - VV
Age
Pack years
Volume
GTVp - glszm - LAHGLE
GTVp - glszm - LALGLE
GTVp - glszm - ZV
GTVp - glszm - LAE
GTVp - glrlm - LRHGLE
GTVp - glrlm - RV
GTVp - glrlm - GLNUN
GTVp - glszm - GLNUN
HR=2.098
HR=1.890
HR=1.440
HR=1.376
HR=1.356
HR=1.355
HR=1.328
HR=1.322
HR=1.322
HR=1.322
HR=1.311
HR=1.301
HR=1.300
HR=1.298
HR=1.291
HR=1.291
HR=1.291
HR=1.285
HR=1.260
HR=1.211
HR=1.162
HR=1.155
HR=1.146
HR=1.146
HR=1.063
HR=1.013
HR=1.004
HR=0.907
HR=0.900
HR=0.894
HR=0.894
HR=0.890
HR=0.877
HR=0.744
HR=0.705
0
1
2
3

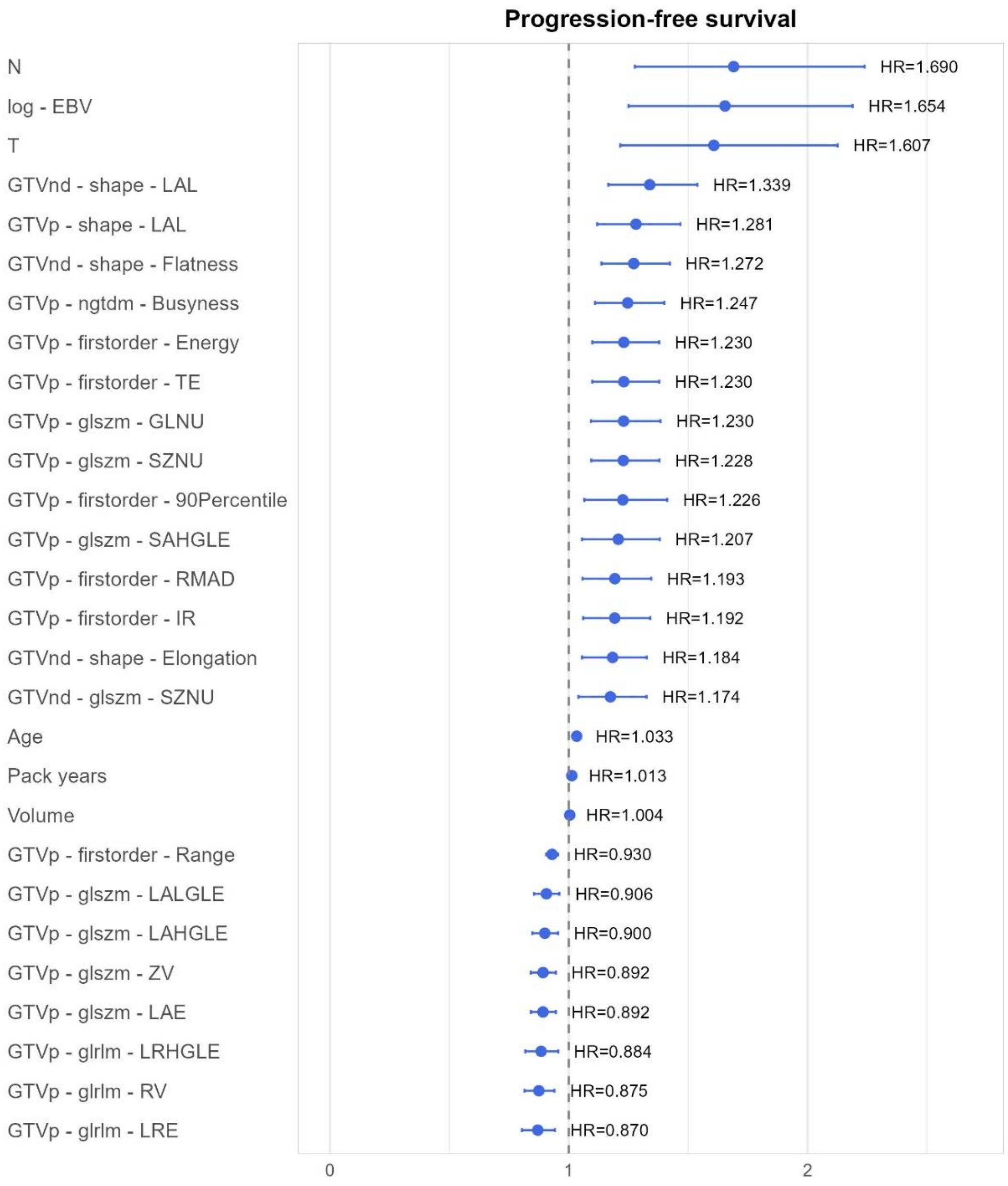
Progression-free survival
N
log - EBV
T
GTVnd - shape - LAL
GTVp - shape - LAL
GTVnd - shape - Flatness
GTVp - ngtdm - Busyness
GTVp - firstorder - Energy
GTVp - firstorder - TE
GTVp - glszm - GLNU
GTVp - glszm - SZNU
GTVp - firstorder - 90Percentile
GTVp - glszm - SAHGLE
GTVp - firstorder - RMAD
GTVp - firstorder - IR
GTVnd - shape - Elongation
GTVnd - glszm - SZNU
Age
Pack years
Volume
GTVp - firstorder - Range
GTVp - glszm - LALGLE
GTVp - glszm - LAHGLE
GTVp - glszm - ZV
GTVp - glszm - LAE
GTVp - glrlm - LRHGLE
GTVp - glrlm - RV
GTVp - glrlm - LRE
HR=1.690
HR=1.654
HR=1.607
HR=1.339
HR=1.281
HR=1.272
HR=1.247
HR=1.230
HR=1.230
HR=1.230
HR=1.228
HR=1.226
HR=1.207
HR=1.193
HR=1.192
HR=1.184
HR=1.174
HR=1.033
HR=1.013
HR=1.004
HR=0.930
HR=0.906
HR=0.900
HR=0.892
HR=0.892
HR=0.884
HR=0.875
HR=0.870
0
1
2

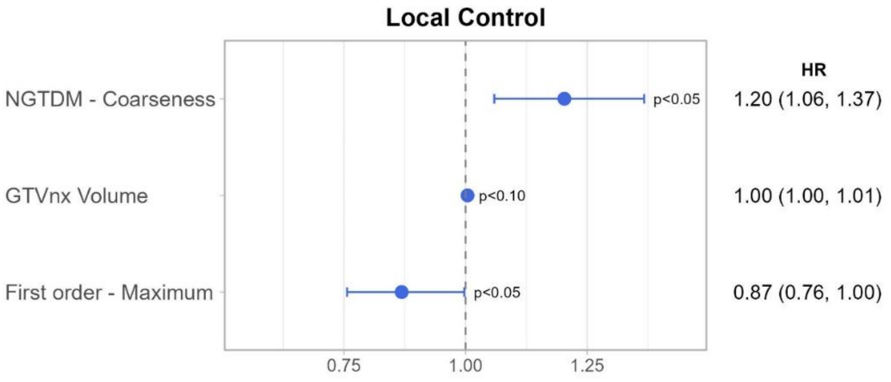
Local Control
NGTDM - Coarseness
GTVnx Volume
First order - Maximum
p<0.05
p<0.10
p<0.05
HR
1.20 (1.06, 1.37)
1.00 (1.00, 1.01)
0.87 (0.76, 1.00)
0.75
1.00
1.25

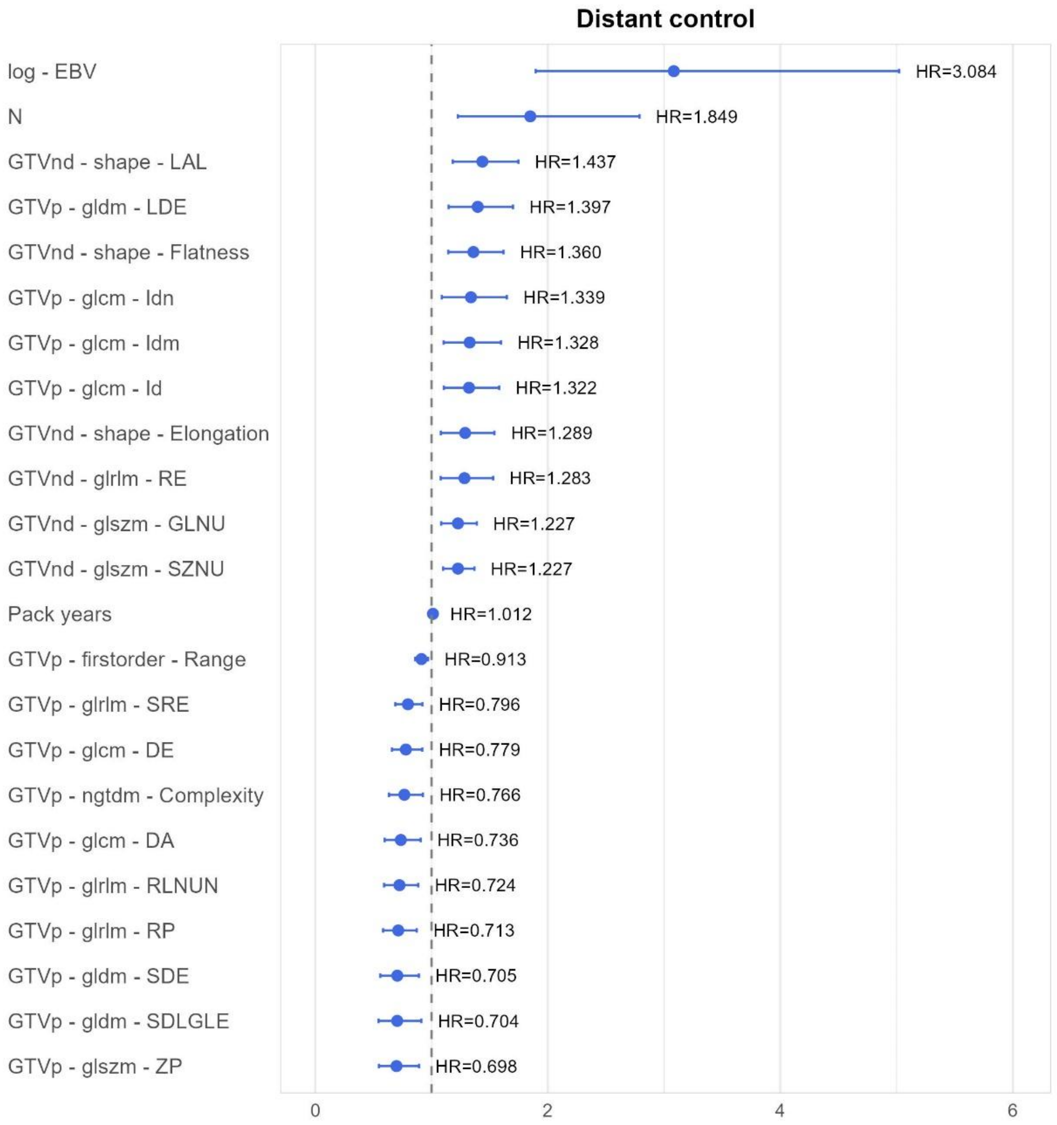

Figure S2. Univariable analysis of radiomic features: The forest plot reports the hazard ratios (HRs) with 95% confidence intervals (CIs) of features with significance levels below 0.01 (for OS, PFS, and DC). For LC, there were no predictors with p<0.01, so the predictors with p<0.1 are displayed instead.

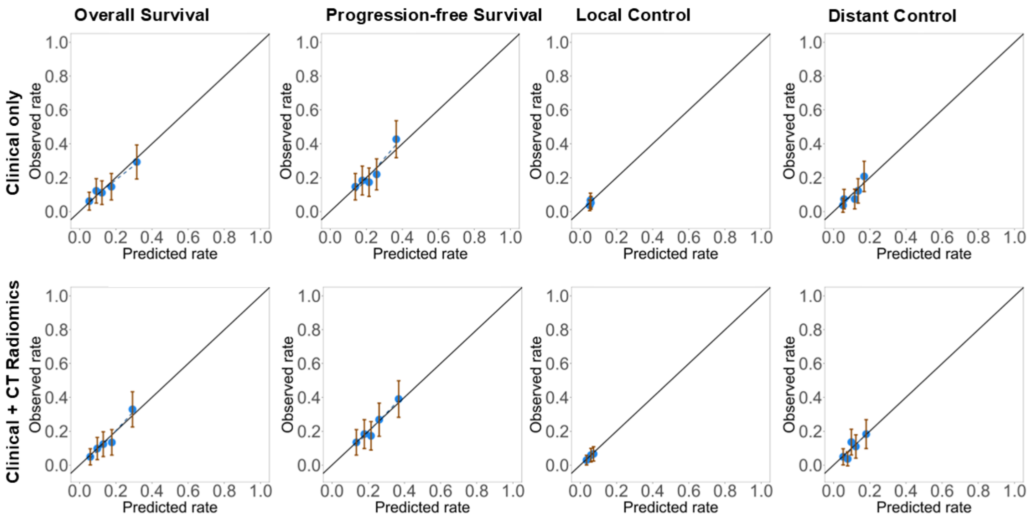

(a)

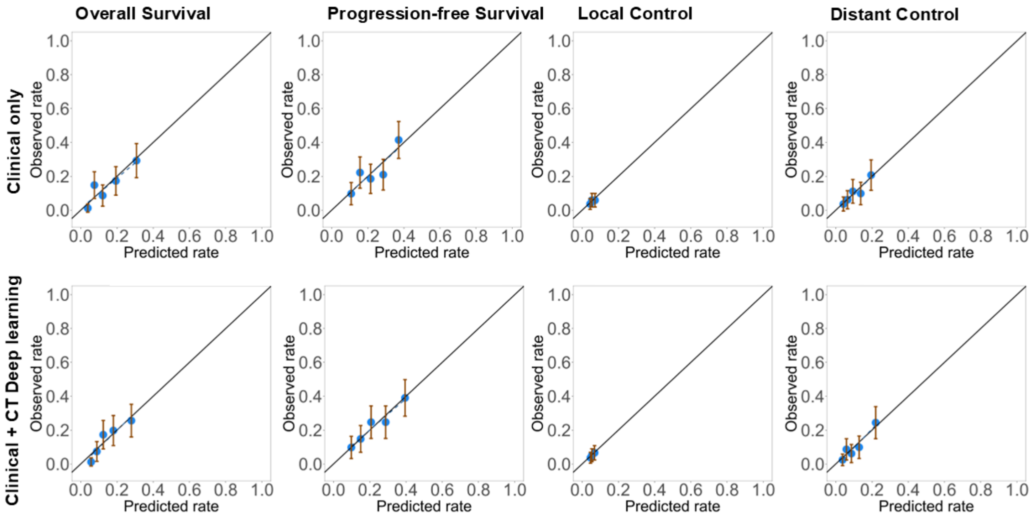

(b)

Figure S3. Calibration plot of the radiomics-based models (a) and the deep learning models (b) of clinical only and clinical + CT for each endpoint.

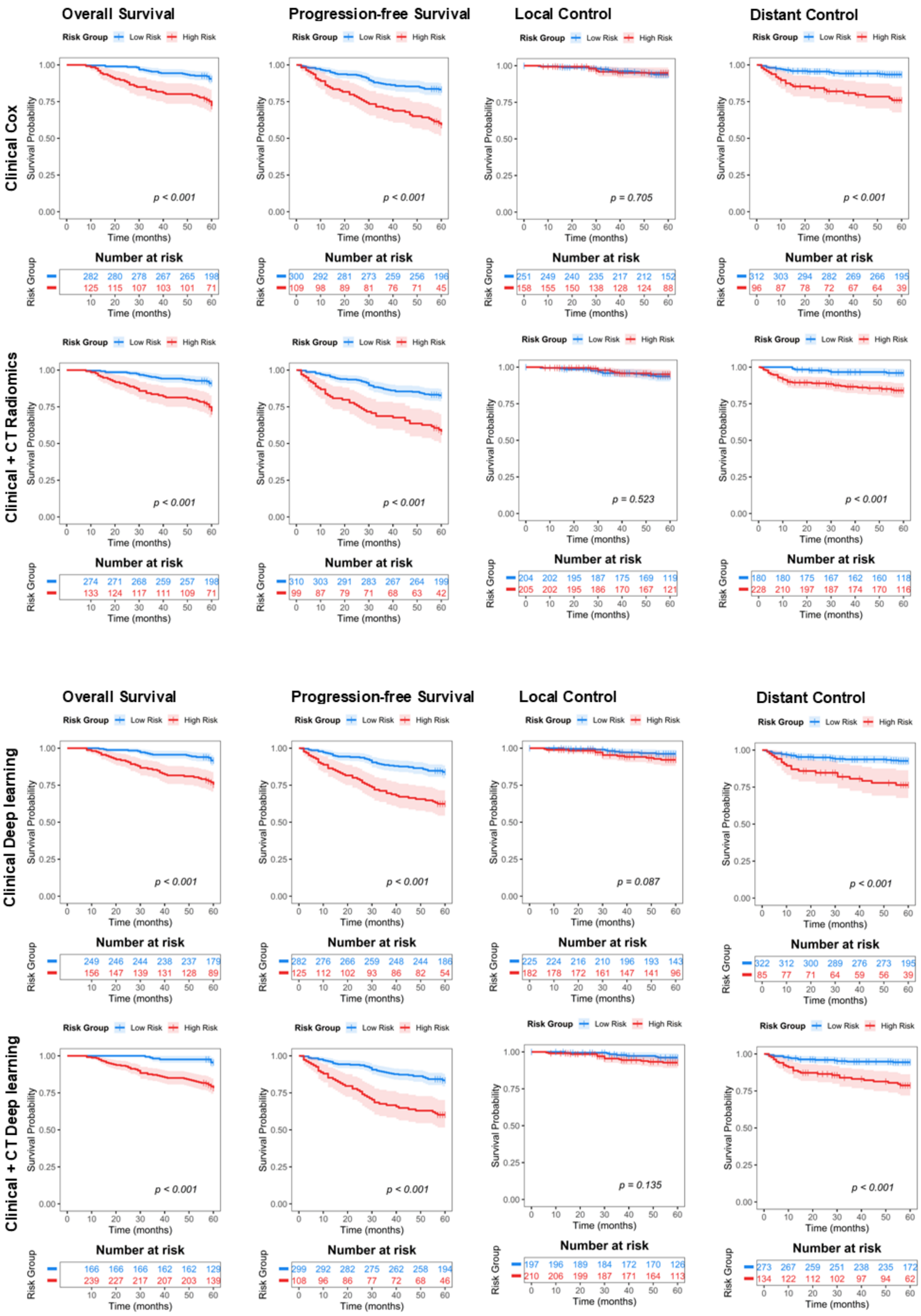

Figure S4. Kaplan-Meier curve at 60th month on the test set.

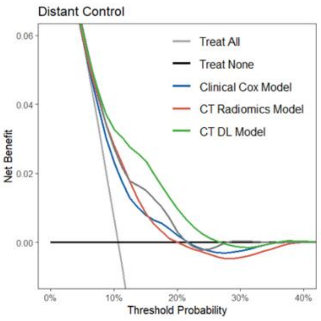

Figure S5. Decision curve analysis results of 60th month on clinical Cox model, CT radiomics model and CT deep learning model, net benefit across decision threshold in the test set.

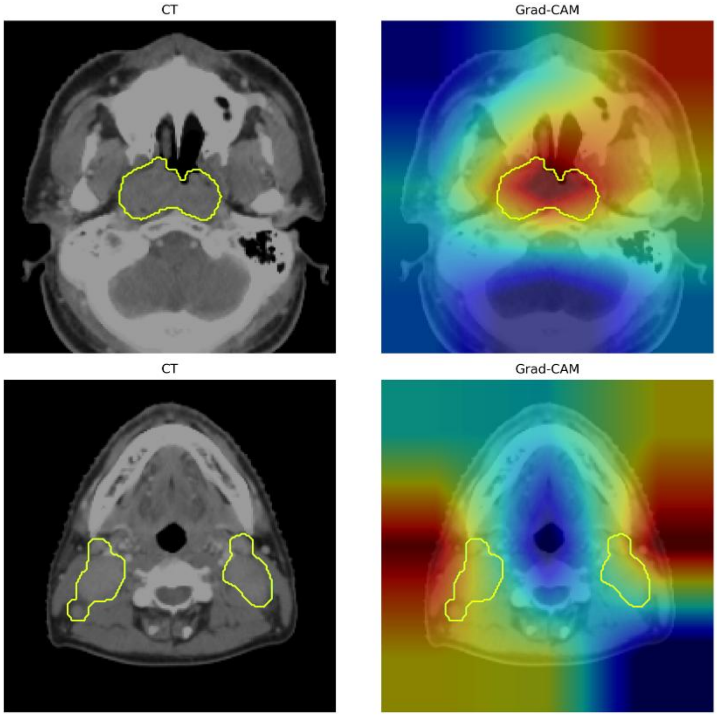

Figure S6. Example of Grad-CAM results of DL models for PFS prediction.

Table S1.1. Radiomics (Cox) model configurations and test performance. Three **selection strategies** were examined: Forced (pre-specified clinical covariates retained; radiomic features additionally selected), Unbiased (clinical + radiomic features competed on equal terms in the same selection pool), and Radiomics-only (no clinical covariates). For each strategy, radiomics features were extracted with GTVp, GTVn, and GTVmulti as **inputs**. **Model parameters** included in the final multivariable model are indicated. **C-index** (95% CI) on the test set for multivariable Cox models. Bold rows denote the best performing configuration for the given endpoint and those results are also shown in Table 2.

Endpoints	Selection strategy	Inputs	Model parameter	C-index (95% CI)
OS	Clinical	Cl	Age, T, N, EBV	0.68 (0.62, 0.75)
	Forced	Cl + GTVp	Age, T, N, EBV, GTVp-GLSZM-SAHGLE	0.69 (0.62, 0.76)
		Cl + GTVn	Age, T, N, EBV, GTVn-shape-MDR	0.69 (0.62, 0.76)
		Cl + GTVmulti	Age, T, N, EBV, GTVp-GLSZM-GLNU, GTVn-shape-MDR	0.67 (0.60, 0.74)
	Unbiased	Cl + GTVp	Age, N, GTVp-GLSZM-GLNU	0.66 (0.59, 0.73)
		Cl + GTVn	Age, N, GTVn-shape-MDR	0.68 (0.61, 0.75)
		Cl + GTVmulti	Age, N, GTVp-GLSZM-GLNUN, GTVn-shape-LAL	0.66 (0.59, 0.73)
	Radiomics	GTVp	GTVp-GLSZM-GLNUN, GTVp-Firstorder-Skewness	0.56 (0.49, 0.63)
		GTVn	GTVn-shape-LAL	0.52 (0.45, 0.60)
		GTVmulti	GTVp-shape-LAL, GTVp-GLSZM-GLNU	0.56 (0.49, 0.63)
PFS	Clinical	Cl	Age, N, GTVp-Volume, Pack-years	0.63 (0.57, 0.69)
	Forced	Cl + GTVp	Age, N, GTVp-Volume, Pack-years, GTVp-NGTDM-Busyness	0.63 (0.57, 0.69)
		Cl + GTVn	Age, N, GTVp-Volume, Pack-years, GTVn-shape flatness	0.63 (0.57, 0.68)
		Cl + GTVmulti	Age, N, GTVp-Volume, Pack-years, GTVp-shape-LAL, GTVn-shape-LAL	0.64 (0.58, 0.69)
	Unbiased	Cl + GTVp	Age, Pack-years, GTVp-NGTDM-Busyness	0.60 (0.55, 0.66)
		Cl + GTVn	Age, Pack-years, GTVn-shape-LAL	0.59 (0.54, 0.65)
		Cl + GTVmulti	Age, Pack-years, N, GTVp-shape-LAL, GTVn-shape-LAL	0.63 (0.58, 0.69)

	Radiomics	GTVp	GTVp-shape NGTDM-Busyness, GTVp-shape sphericity	0.61 (0.55, 0.67)
		GTVn	GTVn-shape-LAL, GTVn-GLSZM-SZNU	0.59 (0.53, 0.65)
		GTVmulti	GTVp-shape-LAL, GTVn-shape-LAL, GTVp-firstorder-Max	0.61 (0.55, 0.67)
LC	Clinical	Cl	GTVp-Volume	0.51 (0.39, 0.62)
	Forced	Cl + GTVp	GTVp-Volume, GTVp-NGTDM coarseness	0.60 (0.48, 0.73)
		Cl + GTVn	GTVp-Volume, GTVn-GLRLM-GLNU	0.50 (0.39, 0.60)
		Cl + GTVmulti*	GTVp-Volume, GTVp-NGTDM coarseness	0.60 (0.48, 0.73)
	Unbiased	Cl + GTVp	GTVp-Volume, GTVp-NGTDM coarseness	0.60 (0.48, 0.73)
		Cl + GTVn	N, GTVn-GLRLM-GLNU	0.51 (0.39, 0.62)
		Cl + GTVmulti*	Volume, GTVp-NGTDM coarseness	0.60 (0.48, 0.73)
	Radiomics	GTVp	GTVp-NGTDM coarseness	0.53 (0.42, 0.64)
		GTVn	GTVn-GLRLM-GLNU	0.52 (0.41, 0.63)
		GTVmulti*	GTVp-NGTDM coarseness	0.53 (0.42, 0.64)
DC	Clinical	Cl	N, Volume, EBV	0.64 (0.57, 0.69)
	Forced	Cl + GTVp	N, Volume, EBV, GTVp-shape flatness	0.65 (0.55, 0.75)
		Cl + GTVn	N, Volume, EBV, GTVn-GLSZM-GLNU	0.63 (0.53, 0.73)
		Cl + GTVmulti	N, Volume, EBV, GTVn-GLSZM-GLNU, GTVn-shape-LAL, GTVp-firstorder-Max	0.64 (0.54, 0.74)
	Unbiased	Cl + GTVp	EBV, GTVp-GLDM-LDE, N	0.62 (0.52, 0.72)
		Cl + GTVn	EBV, GTVn-shape-LAL, GTVn-GLSZM-SZNUN	0.62 (0.51, 0.73)
		Cl + GTVmulti	EBV, GTVn-shape-LAL, GTVp-GLSZM-GLV	0.61 (0.51, 0.72)
	Radiomics	GTVp	GTVp-GLDM-LDE	0.56 (0.47, 0.65)
		GTVn	GTVn-shape-LAL, GTVn-GLSZM-SZNUN, GTVn-GLSZM-GLNU	0.63 (0.55, 0.72)
		GTVmulti	GTVn-shape-LAL, GTVp-GLSZM-ZP	0.64 (0.56, 0.72)

* In the GTVmulti setting of LC model, feature selection identified only GTVp features, no GTVn features were retained.

Table S1.2. Deep learning model configurations and test performance. Each row corresponds to one trained model evaluated on the same test data: FCN (clinical-only) and CT-based CNNs (ResNet18, DenseNet121) with different input parameters. **Inputs** include GTVp (primary tumour), GTVtot (clinical plus CT of primary tumour and lymph nodes as one structure), GTVmulti (clinical plus CT with primary and nodes as separate channels), combined with clinical variables respectively. Bold indicates, for each endpoint, the best deep learning model; the corresponding entry is reproduced in Table 2.

Endpoints	Models	Inputs	C-index (95% CI)
**OS**	FCN	Clinical	0.70 (0.62, 0.75)
	CT-based ResNet18	Clinical + GTVp	0.67 (0.60, 0.74)
		GTVp	0.59 (0.52, 0.67)
		Clinical + GTVtot	0.68 (0.62, 0.74)
		GTVtot	0.64 (0.57, 0.71)
		Clinical + GTVmulti	0.69 (0.63, 0.75)
		GTVmulti	0.64 (0.57, 0.70)
	CT-based DenseNet121	Clinical + GTVp	0.68 (0.62, 0.74)
		GTVp	0.60 (0.53, 0.68)
		Clinical + GTVtot	0.69 (0.62, 0.74)
		GTVtot	0.64 (0.58, 0.70)
		Clinical + GTVmulti	0.69 (0.63, 0.75)
		GTVmulti	0.63 (0.57, 0.71)
**PFS**	FCN	Clinical	0.64 (0.58, 0.70)
	CT-based ResNet18	Clinical + GTVp	0.65 (0.59, 0.70)
		GTVp	0.62 (0.57, 0.69)
		Clinical + GTVtot	0.64 (0.58, 0.69)
		GTVtot	0.65 (0.60, 0.70)
		Clinical + GTVmulti	0.66 (0.60, 0.71)
		GTVmulti	0.65 (0.60, 0.70)
	CT-based DenseNet121	Clinical + GTVp	0.63 (0.58, 0.68)
		GTVp	0.60 (0.55, 0.66)
		Clinical + GTVtot	0.65 (0.59, 0.70)
		GTVtot	0.62 (0.57, 0.68)
		Clinical + GTVmulti	0.65 (0.59, 0.70)
		GTVmulti	0.64 (0.59, 0.70)
	FCN	Clinical	0.55 (0.40, 0.66)

**LC**	CT-based ResNet18	Clinical + GTVp	0.53 (0.40, 0.65)
		GTVp	0.51 (0.40, 0.65)
		Clinical + GTVtot	0.49 (0.35, 0.61)
		GTVtot	0.49 (0.39, 0.62)
		Clinical + GTVmulti	0.54 (0.41, 0.66)
		GTVmulti	0.51 (0.42, 0.63)
	CT-based DenseNet121	Clinical + GTVp	0.51 (0.37, 0.63)
		GTVp	0.50 (0.40, 0.62)
		Clinical + GTVtot	0.47 (0.36, 0.57)
		GTVtot	0.47 (0.36, 0.60)
		Clinical + GTVmulti	0.51 (0.39, 0.63)
		GTVmulti	0.52 (0.42, 0.66)
**DC**	FCN	Clinical	0.66 (0.56, 0.73)
	CT-based ResNet18	Clinical + GTVp	0.66 (0.57, 0.73)
		GTVp	0.63 (0.56, 0.71)
		Clinical + GTVtot	0.66 (0.58, 0.74)
		GTVtot	0.65 (0.58, 0.73)
		Clinical + GTVmulti	0.67 (0.58, 0.74)
		GTVmulti	0.66 (0.58, 0.75)
	CT-based DenseNet121	Clinical + GTVp	0.64 (0.55, 0.70)
		GTVp	0.58 (0.51, 0.67)
		Clinical + GTVtot	0.68 (0.59, 0.76)
		GTVtot	0.66 (0.58, 0.74)
		Clinical + GTVmulti	0.67 (0.59, 0.74)
		GTVmulti	0.65 (0.57, 0.73)

Table S2. Selection frequency of selected features across 1,000 bootstrap forward-selection runs. Results are shown for frequency of features under three settings: Rad-only S2.1, Forced S2.2 and Unbiased S2.3. For each setting the table for GTVp, GTVn and GTVmulti are provided. FreqStep1–3 report how many times a feature was selected as 1st, 2nd or 3rd feature in the forward selection process; TotalFreq = FreqStep1 + FreqStep2 + FreqStep3. The best-performing configuration for each endpoint is indicated.

S2.1 Rad-only

OS GTVp

site	category	feature	FreqStep1	FreqStep2	FreqStep3	TotalFreq
GTVp	shape	Sphericity	10	234	81	325
GTVp	glszm	GrayLevelNonUniformity	242	51	12	305
GTVp	glszm	SmallAreaHighGrayLevelEmphasis	174	39	58	271
GTVp	glszm	GrayLevelNonUniformityNormalized	121	38	13	172
GTVp	ngtdm	Strength	134	9	11	154
GTVp	firstorder	Skewness	31	77	33	141
GTVp	gldm	LargeDependenceHighGrayLevelEmphasis	1	87	48	136
GTVp	glrlm	GrayLevelNonUniformityNormalized	58	26	43	127
GTVp	shape	LeastAxisLength	102	5	19	126
GTVp	glszm	ZoneEntropy	56	20	16	92

OS GTVn

site	category	feature	FreqStep1	FreqStep2	FreqStep3	TotalFreq
GTVn	shape	LeastAxisLength	487	41	9	537
GTVn	shape	Flatness	365	84	34	483
GTVn	glszm	SizeZoneNonUniformity	18	192	39	249
GTVn	shape	Maximum2DDiameterRow	103	76	21	200
GTVn	glrlm	RunEntropy	2	77	65	144
GTVn	shape	Sphericity	0	65	25	90
GTVn	shape	Elongation	21	35	12	68
GTVn	glszm	SizeZoneNonUniformityNormalized	0	33	18	51
GTVn	shape	VoxelVolume	2	10	23	35
GTVn	glszm	SmallAreaEmphasis	0	6	3	9

OS GTV multi

site	category	feature	FreqStep1	FreqStep2	FreqStep3	TotalFreq

GTVn	shape	LeastAxisLength	340	122	22	484
GTVn	shape	Flatness	209	165	81	455
GTVp	glszm	GrayLevelNonUniformity	96	137	47	280
GTVp	glszm	GrayLevelNonUniformityNormalized	59	88	28	175
GTVp	glszm	SmallAreaHighGrayLevelEmphasis	80	79	12	171
GTVn	shape	Maximum2DDiameterRow	54	48	57	159
GTVn	glszm	SizeZoneNonUniformity	8	32	115	155
GTVp	firstorder	Skewness	4	47	99	150
GTVp	shape	LeastAxisLength	46	55	5	106
GTVp	ngtdm	Strength	53	30	14	97

PFS GTVp

site	category	feature	FreqStep1	FreqStep2	FreqStep3	TotalFreq
GTVp	shape	Sphericity	38	229	198	465
GTVp	firstorder	Maximum	78	230	101	409
GTVp	ngtdm	Busyness	295	34	12	341
GTVp	shape	LeastAxisLength	263	32	14	309
GTVp	glszm	GrayLevelVariance	66	62	79	207
GTVp	firstorder	RobustMeanAbsoluteDeviation	72	58	57	187
GTVp	firstorder	Range	6	62	52	120
GTVp	ngtdm	Strength	8	57	35	100
GTVp	firstorder	90Percentile	55	9	7	71
GTVp	glrlm	GrayLevelNonUniformityNormalized	22	23	25	70

PFS GTVn

site	category	feature	FreqStep1	FreqStep2	FreqStep3	TotalFreq
GTVn	shape	LeastAxisLength	604	54	5	663
GTVn	glszm	SizeZoneNonUniformity	106	264	36	406
GTVn	shape	Flatness	230	51	9	290
GTVn	shape	Sphericity	0	67	64	131
GTVn	glszm	SizeZoneNonUniformityNormalized	0	65	31	96
GTVn	shape	Maximum2DDiameterRow	47	28	14	89
GTVn	glrlm	RunEntropy	2	16	10	28
GTVn	shape	Elongation	5	15	6	26
GTVn	shape	VoxelVolume	3	8	15	26
GTVn	glszm	SmallAreaEmphasis	1	4	6	11

PFS GTV multi

site	category	feature	FreqStep1	FreqStep2	FreqStep3	TotalFreq
GTVn	shape	LeastAxisLength	435	94	34	563
GTVp	shape	LeastAxisLength	110	158	49	317
GTVp	firstorder	Maximum	13	114	176	303
GTVn	shape	Flatness	139	101	41	281
GTVp	ngtdm	Busyness	84	139	55	278
GTVn	glszm	SizeZoneNonUniformity	76	76	101	253
GTVp	firstorder	RobustMeanAbsoluteDeviation	21	77	41	139
GTVp	shape	Sphericity	1	22	83	106
GTVp	glszm	GrayLevelVariance	18	47	34	99
GTVn	shape	Maximum2DDiameterRow	45	24	15	84

LC GTVp

site	category	feature	FreqStep1	FreqStep2	FreqStep3	TotalFreq
GTVp						
GTVp	ngtdm	Coarseness	314	75	15	404
GTVp	shape	LeastAxisLength	106	181	37	324
GTVp	glszm	GrayLevelNonUniformityNormalized	61	94	26	181
GTVp	glszm	LowGrayLevelZoneEmphasis	39	38	45	122
GTVp	firstorder	InterquartileRange	68	20	7	95
GTVp	firstorder	Maximum	60	22	4	86
GTVp	shape	Elongation	32	24	24	80
GTVp	glszm	HighGrayLevelZoneEmphasis	0	30	48	78
GTVp	shape	Flatness	6	4	44	54
GTVp	glcm	Idm	43	4	4	51

LC GTVn

site	category	feature	FreqStep1	FreqStep2	FreqStep3	TotalFreq
GTVn	glrlm	GrayLevelNonUniformity	47	47	15	109
GTVn	glszm	SizeZoneNonUniformityNormalized	79	14	13	106
GTVn	shape	LeastAxisLength	44	30	9	83
GTVn	gldm	DependenceNonUniformityNormalized	31	14	8	53
GTVn	shape	Maximum2DDiameterRow	17	17	15	49
GTVn	shape	Elongation	30	7	1	38
GTVn	shape	Sphericity	19	6	6	31
GTVn	shape	VoxelVolume	20	3	7	30
GTVn	shape	MajorAxisLength	20	0	3	23
GTVn	glszm	SmallAreaEmphasis	11	4	0	15

LC GTV multi

site	category	feature	FreqStep1	FreqStep2	FreqStep3	TotalFreq
GTVp	ngtdm	Coarseness	314	85	25	424
GTVp	shape	LeastAxisLength	105	176	40	321
GTVp	glszm	GrayLevelNonUniformityNormalized	39	116	13	168
GTVp	glszm	LowGrayLevelZoneEmphasis	30	0	59	89
GTVp	firstorder	Maximum	61	11	5	77
GTVp	shape	Elongation	25	14	34	75
GTVn	glszm	SizeZoneNonUniformityNormalized	25	31	15	71
GTVn	gldm	DependenceNonUniformityNormalized	16	31	15	62
GTVp	firstorder	InterquartileRange	25	17	9	51
GTVp	glcm	Idm	42	8	0	50

DC GTVp

site	category	feature	FreqStep1	FreqStep2	FreqStep3	TotalFreq
GTVp	glszm	ZonePercentage	441	1	9	451
GTVp	firstorder	Maximum	3	335	79	417
GTVp	glszm	GrayLevelVariance	13	135	178	326
GTVp	glcm	InverseVariance	26	134	57	217
GTVp	glcm	ClusterShade	160	9	10	179
GTVp	ngtdm	Coarseness	98	22	22	142
GTVp	shape	Sphericity	45	32	56	133
GTVp	firstorder	Range	11	42	62	115
GTVp	glszm	HighGrayLevelZoneEmphasis	93	3	6	102
GTVp	ngtdm	Busyness	55	20	25	100

DC GTVn

site	category	feature	FreqStep1	FreqStep2	FreqStep3	TotalFreq
GTVn	shape	LeastAxisLength	454	72	59	585
GTVn	glszm	GrayLevelNonUniformity	169	192	144	505
GTVn	glszm	SizeZoneNonUniformityNormalized	24	293	139	456
GTVn	shape	Flatness	210	40	3	253
GTVn	glrlm	RunEntropy	86	40	8	134
GTVn	shape	Sphericity	0	57	51	108
GTVn	shape	Elongation	41	21	2	64
GTVn	shape	MeshVolume	5	6	20	31
GTVn	shape	Maximum2DDiameterColumn	9	6	5	20
GTVn	glszm	SmallAreaEmphasis	1	3	9	13

DC GTV multi (best performing setting)

site	category	feature	FreqStep1	FreqStep2	FreqStep3	TotalFreq
**GTVn**	**shape**	**LeastAxisLength**	309	135	53	497
GTVn	glszm	GrayLevelNonUniformity	134	93	113	340
**GTVp**	**glszm**	**ZonePercentage**	124	140	51	315
GTVn	shape	Flatness	144	63	32	239
**GTVp**	**firstorder**	**Maximum**	15	71	128	214
GTVp	glszm	GrayLevelVariance	64	68	45	177
GTVn	glszm	SizeZoneNonUniformityNormalized	3	76	88	167
GTVp	glcm	InverseVariance	30	76	47	153
GTVp	glcm	ClusterShade	21	51	53	125
GTVn	glrlm	RunEntropy	62	24	15	101

S2.2 Forced

OS GTVp

site	category	feature	FreqStep1	FreqStep2	FreqStep3	TotalFreq
GTVp	shape	Sphericity	119	108	90	317
GTVp	glszm	SmallAreaHighGrayLevelEmphasis	26	138	42	206
GTVp	glszm	GrayLevelNonUniformityNormalized	5	164	26	195
GTVp	gldm	LargeDependenceHighGrayLevelEmphasis	162	1	15	178
GTVp	shape	Flatness	102	13	62	177
GTVp	glszm	GrayLevelNonUniformity	68	66	11	145
GTVp	firstorder	Skewness	84	15	13	112
GTVp	glcm	InverseVariance	98	6	7	111
GTVp	firstorder	RobustMeanAbsoluteDeviation	47	39	23	109
GTVp	glszm	ZoneEntropy	21	75	13	109

OS GTVn

site	category	feature	FreqStep1	FreqStep2	FreqStep3	TotalFreq
GTVn	shape	LeastAxisLength	612	35	6	653
GTVn	shape	Flatness	11	398	50	459
GTVn	shape	Maximum2DDiameterRow	320	2	7	329
GTVn	glrlm	RunEntropy	7	60	165	232
GTVn	glszm	SizeZoneNonUniformityNormalized	0	107	31	138
GTVn	glszm	SizeZoneNonUniformity	0	71	47	118
GTVn	shape	Elongation	36	23	25	84
GTVn	shape	VoxelVolume	14	7	20	41
GTVn	shape	Sphericity	0	31	7	38
GTVn	glszm	SmallAreaEmphasis	0	11	9	20

OS GTV multi

site	category	feature	FreqStep1	FreqStep2	FreqStep3	TotalFreq
GTVn	shape	LeastAxisLength	641	19	16	676
GTVn	shape	Flatness	10	207	163	380
GTVn	shape	Maximum2DDiameterRow	306	0	7	313
GTVp	glszm	GrayLevelNonUniformityNormalized	0	144	67	211
GTVp	glszm	GrayLevelNonUniformity	0	88	38	126
GTVn	glrlm	RunEntropy	4	31	65	100
GTVp	glszm	ZoneEntropy	0	65	29	94
GTVp	glszm	SmallAreaHighGrayLevelEmphasis	0	65	28	93
GTVn	glszm	SizeZoneNonUniformityNormalized	0	41	41	82
GTVp	shape	Elongation	0	42	28	70

PFS GTVp

site	category	feature	FreqStep1	FreqStep2	FreqStep3	TotalFreq
GTVp	ngtdm	Busyness	547	4	4	555
GTVp	firstorder	Maximum	0	390	91	481
GTVp	shape	Sphericity	15	114	166	295
GTVp	shape	LeastAxisLength	268	7	9	284
GTVp	glszm	GrayLevelVariance	18	55	109	182
GTVp	firstorder	RobustMeanAbsoluteDeviation	21	77	79	177
GTVp	ngtdm	Strength	5	122	41	168
GTVp	firstorder	90Percentile	54	24	23	101
GTVp	glrlm	LongRunEmphasis	5	31	35	71
GTVp	glszm	SmallAreaHighGrayLevelEmphasis	24	17	20	61

PFS GTVn

site	category	feature	FreqStep1	FreqStep2	FreqStep3	TotalFreq
GTVn	shape	Flatness	4	357	87	448
GTVn	shape	LeastAxisLength	70	302	58	430
GTVn	glszm	SizeZoneNonUniformity	16	239	173	428
GTVn	shape	Maximum2DDiameterRow	372	19	27	418
GTVn	shape	Elongation	164	2	26	192
GTVn	glszm	SizeZoneNonUniformityNormalized	86	16	64	166
GTVn	shape	VoxelVolume	102	5	50	157
GTVn	shape	Sphericity	96	4	16	116
GTVn	glszm	SmallAreaEmphasis	82	1	9	92
GTVn	glrlm	RunEntropy	8	4	36	48

PFS GTV multi (best performing setting)

site	category	feature	FreqStep1	FreqStep2	FreqStep3	TotalFreq
GTVp	ngtdm	Busyness	529	0	3	532
GTVn	shape	Flatness	0	287	103	390
GTVp	firstorder	Maximum	0	140	203	343
GTVn	glszm	SizeZoneNonUniformity	0	184	118	302
GTVn	shape	LeastAxisLength	0	198	72	270
GTVp	shape	LeastAxisLength	259	0	5	264
GTVp	ngtdm	Strength	1	47	91	139
GTVp	firstorder	RobustMeanAbsoluteDeviation	22	25	62	109
GTVp	shape	Sphericity	10	27	61	98
GTVp	firstorder	90Percentile	54	3	12	69

LC GTVp (best performing setting)

site	category	feature	FreqStep1	FreqStep2	FreqStep3	TotalFreq
GTVp	ngtdm	Coarseness	353	64	20	437
GTVp	glszm	LowGrayLevelZoneEmphasis	99	44	56	199
GTVp	glszm	GrayLevelNonUniformityNormalized	40	106	44	190
GTVp	shape	Elongation	72	36	30	138
GTVp	glrlm	LongRunEmphasis	51	62	5	118
GTVp	firstorder	Skewness	37	39	18	94
GTVp	firstorder	InterquartileRange	18	36	19	73
GTVp	shape	LeastAxisLength	8	42	16	66
GTVp	shape	Sphericity	13	29	19	61
GTVp	glszm	HighGrayLevelZoneEmphasis	5	20	35	60

LC GTVn

site	category	Feature	FreqStep1	FreqStep2	FreqStep3	TotalFreq
GTVn	glrlm	GrayLevelNonUniformity	225	63	17	305
GTVn	shape	LeastAxisLength	58	86	19	163
GTVn	glszm	SizeZoneNonUniformityNormalized	83	20	15	118
GTVn	shape	Maximum2DDiameterRow	18	58	21	97
GTVn	gldm	DependenceNonUniformityNormalized	31	24	14	69
GTVn	shape	VoxelVolume	29	15	20	64
GTVn	glszm	SizeZoneNonUniformity	39	11	2	52
GTVn	shape	Sphericity	24	12	12	48
GTVn	shape	MajorAxisLength	35	1	3	39
GTVn	shape	Elongation	28	8	2	38

LC GTV multi

site	category	feature	FreqStep1	FreqStep2	FreqStep3	TotalFreq
GTVp	gldm	SmallDependenceLowGrayLevelEmphasis	225	21	15	261
GTVp	glszm	LowGrayLevelZoneEmphasis	119	62	39	220
GTVp	shape	Elongation	61	52	34	147
GTVp	glszm	GrayLevelNonUniformityNormalized	24	71	47	142
GTVp	firstorder	Maximum	81	25	11	117
GTVp	glrlm	LongRunEmphasis	57	49	7	113
GTVn	glszm	SizeZoneNonUniformityNormalized	31	36	18	85
GTVp	glcm	Idm	62	13	4	79
GTVn	glszm	SizeZoneNonUniformity	19	31	22	72
GTVn	shape	LeastAxisLength	28	22	20	70

DC GTVp

site	category	feature	FreqStep1	FreqStep2	FreqStep3	TotalFreq
GTVp	glszm	ZonePercentage	293	34	21	348
GTVp	glszm	GrayLevelVariance	168	91	78	337
GTVp	firstorder	Maximum	49	218	65	332
GTVp	shape	Sphericity	41	101	126	268
GTVp	firstorder	Range	48	114	53	215
GTVp	glcm	ClusterShade	87	51	27	165
GTVp	glcm	InverseVariance	86	51	25	162
GTVp	ngtdm	Busyness	70	22	14	106
GTVp	glszm	SmallAreaEmphasis	2	34	55	91
GTVp	gldm	DependenceVariance	10	40	38	88

DC GTVn

site	category	feature	FreqStep1	FreqStep2	FreqStep3	TotalFreq
GTVn	glszm	GrayLevelNonUniformity	35	352	81	468
GTVn	glszm	SizeZoneNonUniformityNormalized	1	172	213	386
GTVn	shape	LeastAxisLength	235	79	59	373
GTVn	glrlm	RunEntropy	339	4	9	352
GTVn	shape	Flatness	273	36	10	319
GTVn	shape	Elongation	101	6	3	110
GTVn	shape	MeshVolume	14	24	30	68
GTVn	shape	Sphericity	0	23	20	43
GTVn	shape	Maximum2DDiameterColumn	2	11	9	22
GTVn	glszm	SmallAreaEmphasis	0	5	8	13

DC GTV multi

site	category	feature	FreqStep1	FreqStep2	FreqStep3	TotalFreq
GTVn	glszm	GrayLevelNonUniformity	20	217	88	325
GTVn	shape	LeastAxisLength	174	67	67	308
GTVp	firstorder	Maximum	2	151	115	268
GTVn	glrlm	RunEntropy	258	4	1	263
GTVn	shape	Flatness	198	36	17	251
GTVp	glszm	GrayLevelVariance	1	126	113	240
GTVp	glcm	InverseVariance	4	117	54	175
GTVp	glszm	ZonePercentage	132	16	25	173
GTVn	glszm	SizeZoneNonUniformityNormalized	0	61	112	173
GTVp	firstorder	Range	3	41	54	98

S2.3 Unbiased

OS GTVp

site	category	feature	FreqStep1	FreqStep2	FreqStep3	TotalFreq
Age.at.treatment			934	57	9	1,000
N			26	508	357	891
T			3	91	174	268
GTVp	glszm	SmallAreaHighGrayLevelEmphasis	6	75	70	151
GTVp	glszm	GrayLevelNonUniformity	5	58	69	132
GTVp	glszm	GrayLevelNonUniformityNormalized	7	59	59	125
GTVp	ngtdm	Strength	6	75	41	122
Pack-years			12	27	69	108
GTVp	shape	LeastAxisLength	1	10	25	36
GTVp	shape	Elongation	0	2	31	33

OS GTVn

site	category	feature	FreqStep1	FreqStep2	FreqStep3	TotalFreq
Age			901	92	6	999
T			6	79	398	483
GTVn	shape	LeastAxisLength	46	339	54	439
N			12	215	189	416
GTVn	shape	Flatness	20	147	97	264
GTVn	shape	Maximum2DDiameterRow	5	98	22	125
Volume			0	6	101	107
Pack-years			8	15	58	81
EBV DNA			2	4	27	33
GTVn	shape	Elongation	0	3	10	13

OS GTV multi (best performing setting)

site	category	feature	FreqStep1	FreqStep2	FreqStep3	TotalFreq
Age			887	88	20	995
GTVn	shape	LeastAxisLength	38	290	57	385
N			14	183	159	356
GTVn	shape	Flatness	22	133	89	244
T			0	49	173	222
GTVn	shape	Maximum2DDiameterRow	4	116	31	151
GTVp	glszm	GrayLevelNonUniformity	3	34	81	118
GTVp	glszm	GrayLevelNonUniformityNormalized	4	27	82	113
GTVp	ngtdm	Strength	5	45	55	105
GTVp	glszm	SmallAreaHighGrayLevelEmphasis	2	13	53	68

PFS GTVp

site	category	feature	FreqStep1	FreqStep2	FreqStep3	TotalFreq
Age.at.treatment			279	217	181	677
Pack-years			444	78	48	570
N			73	240	226	539
log			60	89	40	189
GTVp	firstorder	Maximum	4	61	117	182
T			22	56	62	140
GTVp	ngtdm	Busyness	31	51	52	134
GTVp	shape	LeastAxisLength	31	48	42	121
GTVp	firstorder	RobustMeanAbsoluteDeviation	9	32	30	71
GTVp	shape	Sphericity	4	14	37	55

PFS GTVn

site	category	feature	FreqStep1	FreqStep2	FreqStep3	TotalFreq
Age			240	282	215	737
Pack-years			336	89	87	512
GTVn	shape	LeastAxisLength	211	174	55	440
GTVn	shape	Flatness	57	131	59	247
T			28	54	165	247
N			31	98	77	206
GTVn	glszm	SizeZoneNonUniformity	32	70	72	174
Volume			15	29	119	163
EBV DNA			34	30	87	151
GTVn	shape	Maximum2DDiameterRow	14	28	11	53

PFS GTV mult

site	category	feature	FreqStep1	FreqStep2	FreqStep3	TotalFreq
Age			227	262	197	686
Pack-years			318	73	56	447
GTVn	shape	LeastAxisLength	206	151	65	422
GTVn	shape	Flatness	55	104	53	212
N			33	69	68	170
T			19	35	73	127
GTVn	glszm	SizeZoneNonUniformity	23	48	52	123
GTVp	firstorder	Maximum	5	29	82	116
EBV DNA			32	30	51	113
GTVp	shape	LeastAxisLength	27	41	40	108

LC GTVp

site	category	feature	FreqStep1	FreqStep2	FreqStep3	TotalFreq
GTVp	ngtdm	Coarseness	271	100	36	407
Volume			56	90	86	232
N			83	66	49	198
Sex			51	49	46	146
Pack-years			60	44	33	137
BMI			42	50	38	130
Interval			57	42	25	124
GTVp	glszm	GrayLevelNonUniformityNormalized	28	49	41	118
GTVp	shape	LeastAxisLength	25	64	29	118
GTVp	glszm	LowGrayLevelZoneEmphasis	22	31	47	100

LC GTVn

site	category	feature	FreqStep1	FreqStep2	FreqStep3	TotalFreq
N			115	76	50	241
Volume			116	41	31	188
BMI			73	69	34	176
GTVn	glrlm	GrayLevelNonUniformity	25	92	52	169
Sex			81	50	30	161
Pack-years			101	38	19	158
Interval			88	45	17	150
PS.score			39	26	30	95
GTVn	glszm	SizeZoneNonUniformityNormalized	41	31	12	84
T			38	25	16	79

LC GTV multi

site	category	feature	FreqStep1	FreqStep2	FreqStep3	TotalFreq
GTVp	ngtdm	Coarseness	218	92	53	363
Volume			62	79	61	202
N			78	51	61	190
Sex			57	41	31	129
BMI			33	54	37	124
Interval			59	35	30	124
Pack-years			52	46	25	123
GTVp	shape	LeastAxisLength	29	59	27	115
GTVp	glszm	GrayLevelNonUniformityNormalized	28	42	41	111
GTVp	glszm	LowGrayLevelZoneEmphasis	21	29	43	93

DC GTVp

site	category	feature	FreqStep1	FreqStep2	FreqStep3	TotalFreq
log			922	46	8	976
GTVp	firstorder	Maximum	5	164	129	298
GTVp	glszm	GrayLevelVariance	21	142	123	286
Pack-years			9	116	84	209
Sex			2	77	63	142
GTVp	firstorder	Range	0	49	89	138
N			12	64	57	133
GTVp	glszm	ZonePercentage	16	69	40	125
GTVp	glcm	InverseVariance	8	57	41	106
Interval			0	35	52	87

DC GTVn

site	category	feature	FreqStep1	FreqStep2	FreqStep3	TotalFreq
EBV DNA			851	105	21	977
GTVn	shape	LeastAxisLength	65	239	75	379
GTVn	glszm	GrayLevelNonUniformity	26	161	126	313
GTVn	shape	Flatness	31	169	62	262
GTVn	glszm	SizeZoneNonUniformityNormalized	0	41	196	237
Pack-years			10	85	113	208
Interval			1	38	104	143
Sex			0	46	71	117
GTVn	glrlm	RunEntropy	7	33	10	50
GTVn	shape	Elongation	3	27	19	49

DC GTV multi

site	category	feature	FreqStep1	FreqStep2	FreqStep3	TotalFreq
EBV DNA			813	91	42	946
GTVn	shape	LeastAxisLength	67	180	93	340
GTVn	glszm	GrayLevelNonUniformity	33	106	67	206
GTVp	glszm	GrayLevelVariance	21	91	89	201
GTVn	shape	Flatness	15	111	50	176
GTVp	firstorder	Maximum	0	71	92	163
Pack-years			7	52	56	115
GTVn	glszm	SizeZoneNonUniformityNormalized	1	27	81	109
GTVp	glcm	InverseVariance	4	39	58	101
Sex			2	45	48	95

Table S3. Results of stepwise forward selection. The hazard ratio (HR), p-value, and training set C-index for each step are reported in the table.

S3.1 Stepwise Forward Selection (OS Endpoint)

Step	Parameters	Hazard Ratio (95% CI)	p-value	c-index training
Step 1				0.66 (0.62, 0.71)
	Age	20.27 (8.53, 48.16)	<0.01	
Step 2				0.69 (0.65, 0.74)
	Age	23.57 (9.84, 56.44)	<0.01	
	N stage	2.30 (1.63, 3.23)	<0.01	
Step 3				0.70 (0.66, 0.74)
	Age	27.42 (11.28, 66.63)	<0.01	
	N stage	1.97 (1.39, 2.80)	<0.01	
	GTVn-Shape-MDR	1.18 (1.06, 1.30)	<0.01	
Step 4				0.71 (0.67, 0.75)
	Age	28.6 (11.51, 71.03)	<0.01	
	N stage	1.93 (1.36, 2.75)	<0.01	
	GTVn-Shape-MDR	1.20 (1.08, 1.33)	<0.01	
	GTVp-GLSZM-GLNU	1.31 (1.14, 1.50)	<0.01	

S3.2 Stepwise Forward Selection (PFS Endpoint)

Step	Parameters	Hazard Ratio (95% CI)	p-value	c-index training
Step 1				0.64 (0.6, 0.68)
	Age	4.07 (2.01, 8.27)	<0.01	
	N stage	1.70 (1.28, 2.25)	<0.01	
	Pack-years	1.28 (1.12, 1.47)	<0.01	
Step 2				0.64 (0.61, 0.68)
	Age	4.67 (2.28, 9.54)	<0.01	
	N stage	1.42 (1.05, 1.91)	<0.05	
	Pack-years	1.24 (1.08, 1.42)	<0.01	
	GTVn-Shape-LAL	1.26 (1.10, 1.45)	<0.01	
Step 3				0.66 (0.63, 0.7)
	Age	4.67 (2.28, 9.54)	<0.01	
	N stage	1.49 (1.10, 2.01)	<0.01	
	Pack-years	1.21 (1.05, 1.40)	<0.01	
	GTVn-Shape-LAL	1.25 (1.10, 1.43)	<0.01	
	GTVp-Shape-LAL	1.25 (1.09, 1.43)	<0.01	
Step 4				0.67 (0.63, 0.7)
	Age	4.59 (2.25, 9.38)	<0.01	
	N stage	1.50 (1.11, 2.02)	<0.01	
	Pack-years	1.22 (1.06, 1.41)	<0.01	
	GTVn-Shape-LAL	1.15 (0.97, 1.35)	1.1e-01	
	GTVp-Shape-LAL	1.25 (1.09, 1.43)	<0.01	

Step	Parameters	Hazard Ratio (95% CI)	p-value	c-index training
	GTVn-Shape-Flatness	1.15 (0.98, 1.34)	7.7e-02	

S3.3 Stepwise Forward Selection (LC Endpoint)

Step	Parameters	Hazard Ratio (95% CI)	p-value	c-index training
Step 1				0.56 (0.47, 0.65)
	Volume	1.25 (0.97, 1.62)	0.08	
Step 2				0.60 (0.51, 0.68)
	Volume	1.35 (1.08, 1.68)	<0.01	
	GTVp-NGTDM-Coarseness	1.24 (1.11, 1.39)	<0.01	
Step 3				0.60 (0.52, 0.68)
	Volume	1.38 (1.06, 1.79)	<0.05	
	GTVp-NGTDM-Coarseness	1.32 (1.16, 1.49)	<0.01	
	GTVp-GLSZM-GLNUN	0.73 (0.51, 1.06)	0.09	

S3.4 Stepwise Forward Selection (DC Endpoint)

Step	Parameters	Hazard Ratio (95% CI)	p-value	c-index training
Step 1				0.61 (0.56, 0.67)
	GTVn-Shape-LAL	1.44 (1.18, 1.75)	<0.01	
Step 2				0.65 (0.59, 0.70)
	GTVn-Shape-LAL	1.43 (1.18, 1.74)	<0.01	
	GTVp-GLSZM-ZP	0.70 (0.55, 0.90)	<0.01	
Step 3				0.65 (0.59, 0.70)
	GTVn-Shape-LAL	1.37 (1.12, 1.67)	<0.01	
	GTVp-GLSZM-ZP	0.69 (0.54, 0.89)	<0.01	
	GTVn-GLSZM-GLNU	1.16 (1.02, 1.33)	<0.05	

Table S4. The final radiomics model parameters, coefficients, and hazard ratios

Endpoint	Parameters	Coefficients	Hazard ratio (95% CI)	p-value
OS	Age[1]	3.31	27.42 (11.28, 66.63)	<0.01
	N	0.68	1.97 (1.39, 2.80)	<0.01
	GTVn-Shape MDR	0.16	1.18 (1.06, 1.3)	<0.01
PFS	Age	1.54	4.67 (2.28, 9.54)	<0.01
	N	0.40	1.49 (1.1, 2.01)	<0.01
	Pack-years	0.19	1.21 (1.05, 1.4)	<0.01
	GTVn-Shape LAL	0.23	1.25 (1.1, 1.43)	<0.01
	GTVp-Shape LAL	0.22	1.25 (1.09, 1.43)	<0.01
LC	Volume	0.30	1.35 (1.08, 1.68)	<0.01
	GTVp-NGTDM-Coarseness	0.22	1.24 (1.11, 1.39)	<0.01

Endpoint	Parameters	Coefficients	Hazard ratio (95% CI)	p-value
DC	GTVn-Shape LAL	0.36	1.43 (1.18, 1.74)	<0.01
	GTVp-GLSZM ZP	-0.35	0.70 (0.55, 0.90)	<0.01

1. Age was z-score normalised.

Table S5. Discrimination and calibration metrics for local control (LC). Metrics are reported for the clinical-only Cox model and the best radiomics Cox model (GTVp; clinical + CT). Values are presented as estimate (95% confidence interval). C/D AUC = cumulative/dynamic AUC; Uno's C = Uno's concordance index.

Metric	Timepoint	Clinical Model (Estimate)	Clinical Model (95% CI)	Radiomics Model (Estimate)	Radiomics Model (95% CI)
Uno's C-index	60 months	0.51	0.40 - 0.62	0.60	0.48 - 0.72
Brier score	36 months	0.04	0.02 - 0.05	0.04	0.02 - 0.05
	60 months	0.05	0.03 - 0.07	0.05	0.03 - 0.07

Abbreviations:

- 90Percentile: 90th Percentile
- DA: Difference Average
- DC: Distant Control
- DE: Difference Entropy
- EBV: Epstein-Barr Virus
- FCN: Fully Connected Networks
- GLCM: Gray Level Co-occurrence Matrix
- GLDM: Gray Level Dependence Matrix
- GLNU: Gray Level Non-Uniformity
- GLNUN: Gray Level Non-Uniformity Normalized
- GLRLM: Gray Level Run Length Matrix
- GLSZM: Gray Level Size Zone Matrix
- GLV: Gray Level Variance
- GTVmulti: Combined GTVp and GTVn (feature level or dual-channel input)
- GTVn: Gross Tumour Volume – lymph node
- GTVp: Gross Tumour Volume – Primary Tumour
- GTVtot: Total Gross Tumour Volume (primary tumour + lymph nodes as one structure)
- HGLZE: High Gray Level Zone Emphasis
- ID: Inverse Difference
- IDM: Inverse Difference Moment Normalized
- IDN: Inverse Difference Normalized
- IMC2: Informational Measure of Correlation 2
- IR: Interquartile Range
- LAE: Large Area Emphasis
- LAHGLE: Large Area High Gray Level Emphasis
- LAL: Least Axis Length
- LALGLE: Large Area Low Gray Level Emphasis
- LC: Local Control
- LDE: Large Dependence Emphasis
- LRE: Long Run Emphasis
- LRHGLE: Long Run High Gray Level Emphasis
- Max: Maximum Intensity
- MDDR / MDR: Maximum 2D Diameter Row
- MV: Mesh Volume
- N: N Stage
- NGTDM: Neighboring Gray Tone Difference Matrix
- OS: Overall Survival
- PFS: Progression-Free Survival
- Range: Intensity Range
- RE: Run Entropy
- RLNUN: Run Length Non-Uniformity Normalized
- RMAD: Robust Mean Absolute Deviation
- RP: Run Percentage
- RV: Run Variance
- SAHGLE: Small Area High Gray Level Emphasis
- SDE: Small Dependence Emphasis
- SDLGLE: Small Dependence Low Gray Level Emphasis
- SRE: Short Run Emphasis
- SRHGLE: Short Run High Gray Level Emphasis
- SZNU: Size Zone Non-Uniformity
- SZNUN: Size Zone Non-Uniformity Normalized
- T: T Stage
- TE: Total Energy
- VV: Voxel Volume
- ZE: Zone Entropy
- ZP: Zone Percentage
- ZV: Zone Variance
